# Extending the theory of classical nonsolvent-induced phase separation to regulate membrane pores

**DOI:** 10.1093/nsr/nwag306

**Published:** 2026-05-27

**Authors:** Chaoyang Jia, Chenkai Mu, Yiwen Chen, Hongjun Zhang, Willem Verfaillie, Scout Caspers, Ivo F J Vankelecom, Wenjing Lu, Xianfeng Li

**Affiliations:** Dalian Institute of Chemical Physics, Chinese Academy of Sciences, Dalian 116023, China; University of Chinese Academy of Sciences, Beijing 100049, China; Dalian Institute of Chemical Physics, Chinese Academy of Sciences, Dalian 116023, China; University of Chinese Academy of Sciences, Beijing 100049, China; State Key Laboratory of Particle Detection and Electronics, University of Science and Technology of China, Hefei 230026, China; State Key Laboratory of Particle Detection and Electronics, University of Science and Technology of China, Hefei 230026, China; Membrane Technology Group, Centre for Membrane Separations, Adsorption, Catalysis and Spectroscopy for Sustainable Solutions (cMACS), Faculty of Bioscience Engineering, KU Leuven, Leuven 3001, Belgium; Membrane Technology Group, Centre for Membrane Separations, Adsorption, Catalysis and Spectroscopy for Sustainable Solutions (cMACS), Faculty of Bioscience Engineering, KU Leuven, Leuven 3001, Belgium; Membrane Technology Group, Centre for Membrane Separations, Adsorption, Catalysis and Spectroscopy for Sustainable Solutions (cMACS), Faculty of Bioscience Engineering, KU Leuven, Leuven 3001, Belgium; Dalian Institute of Chemical Physics, Chinese Academy of Sciences, Dalian 116023, China; Dalian Institute of Chemical Physics, Chinese Academy of Sciences, Dalian 116023, China

**Keywords:** nonsolvent-induced phase separation, hydrodynamics, nucleation-growth mechanism, membrane structure regulation

## Abstract

Nonsolvent-induced phase separation, employed for over 60 years to prepare porous membranes, still has unclear pore formation mechanisms due to coupled variables. Classical theory links the distinct pore morphologies, i.e. macrovoids or cellular pores, to instantaneous and delayed phase separation, respectively. However, when the formations of macrovoids and cellular pores were decoupled in a tunable device that regulates the nonsolvent hydrodynamics, it was proven that hydrodynamic instability drives macrovoid formation, while cellular pores form via a nucleation-growth mechanism. By establishing a quantitative relationship between nonsolvent and area density of cellular pores, we achieved further optimization of the membrane morphology, enabling its application in vanadium flow batteries with significantly enhanced performance. This work extends the theory of phase separation and provides a causality-driven framework for precision membrane design.

## Teaser

This work decouples the formation of two microstructures in nonsolvent induced phase separation, clarifies the hydrodynamic mechanism behind macrovoid formation, and establishes the correlation between cellular pores and thermodynamic descriptor.

## INTRODUCTION

**Figure 1.**
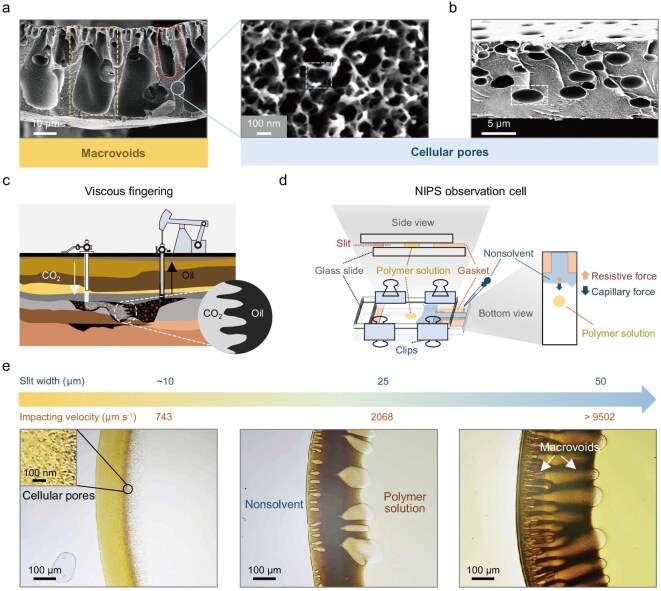
Pore formation mechanism in NIPS. (a) Cross-sectional scanning electron microscopy (SEM) images of the membrane prepared using water as nonsolvent, displaying ‘side-by-side macrovoids’ (yellow box: pear-shaped macrovoids; red box: finger-like macrovoids) with the macrovoid walls containing tightly arranged CPs. (b) Cross-sectional SEM image of the membrane prepared using *n*-hexane as nonsolvent, only containing highly dispersed CPs (white box). (c) Schematic illustration of viscous fingering at the oil/CO_2_ interface in the CO_2_-enhanced oil recovery. (d) The *in-situ* NIPS observation cell (OC). A droplet of polymer solution was sandwiched between two glass slides, creating a round spot (yellow area). Nonsolvent (blue region) was added to the uncovered region of the bottom glass slide and was pulled into the slit between the two glass slides by capillary action. Upon contact of the nonsolvent with the polymer solution, phase separation occurred. The gaskets were copper foils with a certain thickness, sandwiched between the glass slides to adjust the slit width. (e) Structural evolution of polymer precipitation with increasing slit width, as visualized from the OC. Without gaskets (leftmost bottom), a precipitated polymer layer with only CPs was observed. With increasing gasket thickness from 25 to 50 μm, macrovoid formation accompanied by CPs became more obvious. Significantly, the images observed in the OC corresponded well with the morphologies of the actual membranes. Polymer solution: 15 wt.% PBI–DMAc solution. Nonsolvent: water. SEM magnification: (a) left ×1000, right ×50 000; (b) ×2500 .

Nonsolvent-induced phase separation (NIPS) was in fact discovered accidentally by Loeb and Sourirajan in the 1960s [1]. It refers to the contact, mostly via immersion, of a cast polymer solution with a nonsolvent to induce its solidification into a porous membrane (Fig. S1) [2–4]. Typically, the membranes exhibit distinct microstructures (Table S1): macrovoids (e.g. Fig. 1a and Fig. S2) surrounded by cellular pores (CPs) (e.g. Fig. 1b and Fig. S2), or exclusively CPs (e.g. Fig. 1c and Fig. S2). Conventional understanding attributes these structural variations to two distinct phase separation modes: instantaneous demixing generates macrovoids in the matrix, while delayed demixing only yields CPs [5–7]. Although parameters such as solvent–nonsolvent pair selection and polymer concentration enable empirical regulation of membrane microstructures [8–10], often via a trial-and-error approach, a unified theory is still missing to explain the formation of these structures more clearly. Crucially, the concurrent formation of macrovoids and CPs obscures the mechanism study. At present, the commonly used strategies to investigate the membrane formation mechanism in NIPS primarily include *in-situ* microscopy and spectroscopy observations [11–15], and theoretical simulations [16–19] (Supplementary Text). Nonetheless, the macroscopic mass transfer or phase separation information collected from *in-situ* spectroscopy does not directly clarify the microscopic macrovoid or CP formation [11–15]. Phase separation simulations also cannot perfectly predict the exact formation of membrane morphologies [16–19]. Notably, a remarkable phenomenon is that, different from immersion-based NIPS, membranes prepared by other commonly used phase separation approaches (such as vapor or thermally induced phase separation) contain exclusively CPs without macrovoids (Fig. S3 and Tables S1–S3). Thus, it is reasonable to infer that the formation mechanism of CPs differs from that of macrovoids, i.e. they cannot be simultaneously explained within the theoretical framework of phase separation.

We also noticed that multiple macrovoids growing side by side resembled the pattern of viscous fingering observed in CO_2_-enhanced oil recovery [20] (Fig. 1c and Fig. S4), also known as the Saffman-Taylor instability in hydrodynamics [21–23]. Inspired by this, a modified Hele-Shaw cell combined with fluorescence and optical microscopy was assembled for the *in-situ* observation of the NIPS process (Fig. 1d and Fig. S5). Polybenzimidazole (PBI) (Fig. S1) was selected as the model polymer because of its strong relevance in e.g. electrodriven membrane processes [24–26] and distinct optical contrast under microscopy. *N,N*-dimethylacetamide (DMAc) served as solvent, while nonsolvents with varying solubility in DMAc were systematically investigated (Table S4). By using different nonsolvents, distinct membrane microstructures were produced, including side-by-side macrovoids (Fig. 1a) and tightly (Fig. 1a) or dispersedly (Fig. 1b) arranged CPs. The coexistence of macrovoids and CPs combined with their significant structural differences highlights the necessity to decouple their formation processes. The fluid device was thus designed to employ a polymer solution droplet confined between two glass slides, with nonsolvent introduced through capillary action to initiate phase separation (Fig. 1d and Fig. S5). Manipulating the slit width effectively adjusted the interface geometry, which caused the appearance or disappearance of macrovoids, thus decoupling their formation from that of CPs (Fig. 1e and Movies S1–S3).

## RESULTS AND DISCUSSION

### Macrovoid formation mechanism

The finger-like macrovoid pattern is highly similar to the phenomenon of viscous fingering (Fig. 1c and Fig. S4), which describes a finger-like interface created by the displacement of a high-viscosity fluid by a low-viscosity one [23,27]. An *in-situ* NIPS observation cell (OC) (Fig. 1d and Fig. S5) was designed to study macrovoid formation, based on the Hele-Shaw cell (Fig. S4) in hydrodynamic experiments [28–30]. The OC was equipped with a microscope for NIPS observation.

The images observed in the OC (Fig. 1e) corresponded very well with the cross-sectional morphologies of the actual porous membranes (Fig. 1a and b and Fig. S2). During membrane preparation, finger-like macrovoids growing top-to-bottom were gradually blocked by the bottom substrate, resulting in their transformation into pear-shaped macrovoids (Fig. 1a). Without gaskets in OC, the slit width (*d*_L_) between the two glass slides was estimated to be ∼10 μm (Fig. 1e, Figs S6 and S7, and Table S5). Larger *d*_L_ could be controlled by changing the gasket thickness (Fig. 1d). Following capillary transport rules [31], the resistive force decayed more rapidly than the capillary force with increasing *d*_L_ (Fig. S5). An expanded *d*_L_ thus led to a stronger impact by the nonsolvent due to its increased velocity which could also be calculated from microscope movies (Movies S1–S3). The increased nonsolvent impact transformed the pattern from no macrovoids at all to full macrovoids (Fig. 1e). Employing the designed OC thus allowed direct regulation of macrovoid formation by changing the contact states between nonsolvent and polymer solution. These results indicated that macrovoid formation correlates closely with hydrodynamic factors.

**Figure 2.**
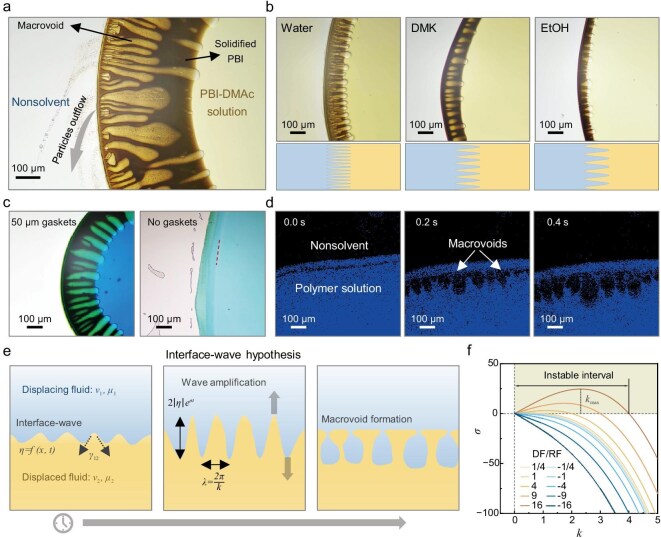
Macrovoid formation mechanism. (a) *In-situ* microscopy observation in OC. Particles turbulence inside the macrovoids and remarkable particle outflow denoted the fluid motion (as clearly visible in Movie S4). These particles are polymer particles washed down by the fluid. (b) Images of macrovoids formed after contacting with different nonsolvents for 20 s and corresponding simulation diagrams of interface-waves. (c) Images of a polymer solution stained by toluidine blue after contacting with nonsolvent (water) for 20 s. The color in macrovoids is lighter, showing the dilution effect of the nonsolvent influx (left). The red dotted line was the frontier of phase separation (right). (d) Fluorescence microscopic images of nonsolvent in contact with a polymer solution for different times. Only the polymer had a fluorescent signal in the system. The weak signal in the macrovoids indicated a significant nonsolvent influx. (e) Schematic diagram of the evolution of instable interface-waves to form macrovoids. If a low-viscosity liquid (viscosity *μ*_1_ and velocity *v*_1_) displaces a high-viscosity liquid (*μ*_2_ and *v*_2_), it is easy to induce an increased amplitude of the interfacial wave, which will evolve into macrovoids. (f) Wavenumber (*k*) dependence of amplification rate (*σ*) of *η*=*f*(*t*) at different driving force (DF)/restraining force (RF) fields. The instable interval denotes that macrovoids are inclined to form. If *σ* > 0, macrovoids form easily, while macrovoids cannot form when *σ* < 0. Generally, if *σ* > 0, increasing DF/RF values can expand the instable *k* interval, increase the most instable wavenumber (*k*_max_), and facilitate macrovoid formation. If DF < 0, *σ* is always less than zero, meaning an always stable interface and no macrovoid formation. Polymer solution: (a–d) 15 wt.% PBI–DMAc solution .

To reinforce this finding, the NIPS process was examined via both optical and fluorescence microscopy experiments. Macrovoids progressively grew from the two-phase boundary to the center of the polymer solution droplet (Fig. 2a). Macrovoid formation, in the water, dimethyl ketone (DMK) and ethanol (EtOH) systems (Fig. 2b), revealed variations in their spatial arrangement dependence. In order to improve the visualization of the nonsolvent distribution, toluidine blue was applied to stain the polymer solution. The clearly lighter color in the macrovoids than in the polymer solution revealed the influx of substantial nonsolvent into the macrovoids within a short time span (Fig. S8). Figure 2c revealed that convection took place in macrovoids (left), thus enhancing mass transfer, whereas only stable diffusion occurred in the system without macrovoids (right). This convection was verified through the particle turbulence within macrovoids (Movie S4 with snapshot in Fig. 2a). These particles were formed by the erosion of precipitated polymer (PBI) owing to convection inside the macrovoids. The remarkable particle outflow into the nonsolvent area indicated the connectivity of the internal and external flow fields (Fig. 2a). In this three-component system, the fluorescence is generated solely by PBI. The fluorescence intensity within macrovoids was notably reduced, further showing a predominance of solvent–nonsolvent mixtures (Fig. 2d, Fig. S9 and Movie S5). In addition, a lower polymer concentration in the cast film also increased the formation probability of the macrovoids (Fig. S10), because insufficient precipitated polymers failed to interconnect the different regions and form an instable interface. All these phenomena are closely related to the hydrodynamic behavior, confirming the aforementioned assumptions.

Saffman-Taylor instability was utilized to correlate macrovoid formation with hydrodynamics [28,32,33]. In the absence of a universal theory for hydrodynamic instability, a theory based on the interface-wave hypothesis, albeit inevitably flawed, was selected to explain macrovoid formation (Fig. 2e and Fig. S11). In Eq. 1, the exponential term (*e^σt^*) of the amplitude is the key to determine whether viscous fingering can appear. If the amplification rate *σ* > 0, the interface-wave amplitude will intensify exponentially over time, yielding an instable wave, thus significantly contributing to macrovoid formation.


(1) \begin{eqnarray*}
\hat{\eta }{\mathrm{ = }}\left| {\hat{\eta }} \right|{e}^{\sigma t}\sin \left( {kx + \theta } \right),
\end{eqnarray*}

(2) \begin{eqnarray*}
\sigma &=& \frac{{\left( {{\mu }_2 - {\mu }_1} \right)vk}}{{{\mu }_1 + {\mu }_2}} - \frac{{\frac{{d_L^2}}{{12}}\gamma {k}^3}}{{{\mu }_1 + {\mu }_2}}\\
&=& {\mathrm{DF}} \times k - {\mathrm{RF}} \times {k}^3,
\end{eqnarray*}

(3) \begin{eqnarray*}
{\lambda }_{\max } = \frac{{2\pi }}{{{k}_{\max }}} = 2\pi \sqrt {\frac{{3{\mathrm{RF}}}}{{{\mathrm{DF}}}}} ,
\end{eqnarray*}

where, $\hat{\,\,\eta }$ is the interface-wave expression, $|\hat{\eta }|$ is the amplitude, *t* is time, *k* is wavenumber*, x* is position and *θ* is phase. *μ*_1_ and *μ*_2_ are the viscosities of the displacing and the displaced liquids, respectively. *v* is the absolute value of the impacting velocity of two liquids, *d*_L_ is the slit width, and *γ* is the interfacial tension of two liquids. *k*_max_ and *λ*_max_ are the most instable wavenumber and wavelength, respectively. The first term constant of Eq. 2 is defined as the driving force (DF) field, and the second term constant is defined as the restraining force (RF) field.

*σ* can be estimated by the *σ*–*k* relation (Eq. 2), which serves as a stability criterion for interface-waves (i.e. *σ* > 0 means instable, *σ* ≤ 0 means stable). If the low-viscosity nonsolvent displaces the high-viscosity polymer solution (*μ*_1_ < *μ*_2_), then DF > 0 and RF > 0. A larger DF/RF will lead to a wider instable interval and higher *σ*, in which macrovoids can form more easily (Fig. 2f). According to Eq. 3, *λ*_max_ in systems of water, DMK and EtOH increased sequentially (Fig. 2b), which is associated with a gradually decreasing DF/RF ratio. Hence, reducing the nonsolvent impact (by lowering its velocity *v*) or increasing *γ* can effectively avert macrovoid formation by stabilizing the interface-wave (lowering *σ*, Fig. 2f). When extending this theory to other polymer, e.g. polyethersulfone (PES), the increased nonsolvent impacting velocity led to macrovoid formation as well (Fig. S12). Furthermore, the displacement of a low-viscosity fluid by a high-viscosity one (*μ*_1_ > *μ*_2_) could yield a stable interface-wave (*σ* < 0) to create a macrovoid-free structure, hence also supporting this theory (Fig. S13 and Movie S6). Concurrently, the interface between phase-separated and phase-unseparated regions might also exhibit instability (Fig. S14), which may arise from fluctuations in the external flow field or the space-squeezing effect of polymer precipitation. As a common hydrodynamic phenomenon, the finger-like interface also appeared in the displacement process of miscible systems (e.g. water and polyethylene glycol 200, Fig. S15). These findings affirm that macrovoid formation should be quite explained by the hydrodynamic instability.

However, the NIPS process challenges the classical Saffman-Taylor theory which was developed for displacement between two immiscible phases, because of its inherently complex evolution involving three components and multiple phases. Thus, other types of instabilities (i.e. convection) may also be considered, such as Marangoni convection driven by interfacial tension gradients from nonsolvent–solvent mixing [34–36], thermocapillary effects induced by temperature gradients from mixing-induced exotherms [37,38], and mechanical disturbances arising from polymer precipitation. Thus, the strategies to suppress macrovoids should focus on controlling convection. For instance, pre-incorporating the nonsolvent into the polymer solution can reduce interfacial tension gradients and mixing exotherms in the NIPS process, thereby decreasing convection to suppress macrovoids [39,40]. Alternatively, increasing polymer solution viscosity [41,42] or pre-solidifying the polymer surface [43,44] can withstand the flow impact to eliminate macrovoids. These approaches underscore the need to model macrovoid formation using hydrodynamic instability theories rather than classical thermodynamic frameworks, as the former better captures the transient fluid behaviors which are critical to macrovoid evolution.

### CP formation mechanism

**Figure 3.**
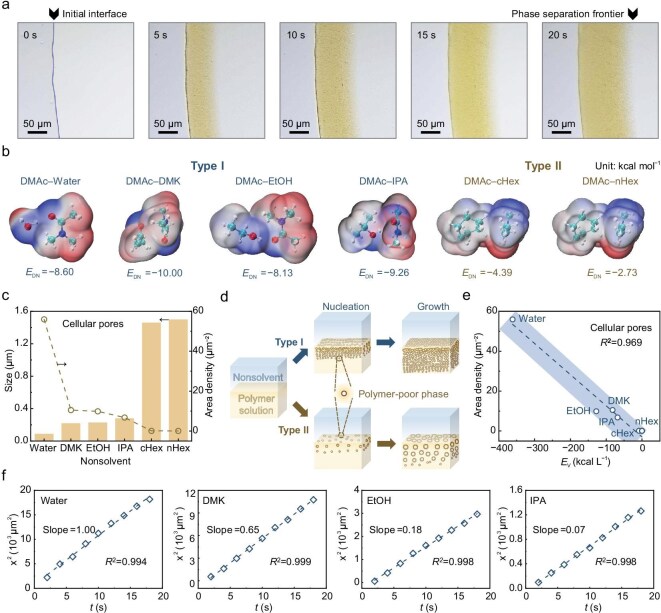
CP formation mechanism. (a) *In-situ* microscopic images of CP formation. The phase separation interface moved over time from the boundary to the inner polymer solution in the OC without gaskets. (b) Electrostatic potential surface and interaction energy (*E*_DN_) of solvent (DMAc) with different nonsolvents, classified as Type I (low *E*_DN_, *δ* > 0.5) and Type II (high *E*_DN_, *δ* < 0.5). (c) Size and area density of CPs in the membranes prepared with different nonsolvents, obtained from cross-sectional SEM images. (d) Schematic diagram of the distinct CP arrangement mechanism in membranes prepared using different nonsolvents. (e) Correlation between the area density of CPs and the thermodynamic descriptor *E*_V_. *E*_V_ contains Δ*E*_NIPS_ and nonsolvent molecular volume. *E*_V_ demonstrates a strong positive correlation with the area density (*R*^2^ = 0.969), confirming its accuracy in predicting membrane microstructures. (f) The plot of the square of the moving distance (*x*^2^) of the phase separation frontier versus time (*t*) in Type I systems. The plots align well with the solution of the relevant Fick’s diffusion equation, demonstrating strong linearity.

CPs always appeared in membranes prepared via phase separation (Tables S1–S3), arranged either dispersedly or tightly. Diverse nonsolvents, varying in miscibility with solvent (DMAc) (Table S4), were used to produce CPs with different arrangements. Quantified by the molar ratio (*δ*) of nonsolvent to DMAc at the cloud points (i.e. critical points of polymer precipitation), these nonsolvents were categorized into two groups: Type I (with *δ* > 0.5), consisting of water, DMK, EtOH and isopropanol (IPA), and Type II (with *δ* < 0.5) with cyclohexane (cHex) and *n*-hexane (nHex) (Table S6). When the gaskets were removed from the OC, solely CPs formed along with the stable movement of the interface upon using Type I nonsolvents (Fig. 3a and Fig. S16). Owing to high volatility and slow nonsolvent–solvent exchange, Type II systems under OC exhibited only CP formation without an obvious polymer precipitation interface. In Type I nonsolvents, the polymer-poor phases (i.e. the CP precursors) closely interconnected to form a sponge-like structure (Fig. 1a and Fig. S2). Conversely, Type II nonsolvents induced well-separated and larger CPs (Fig. 1b and Fig. S2). Focused ion beam-SEM images (Fig. S17) further confirmed that the big pores prepared with Type II nonsolvents were large-size CPs rather than macrovoids, owing to the relatively dense pore walls. Regardless of the nonsolvents used, the initially formed cellular polymer-poor phases served as the dispersed phases, while the polymer-rich phases constituted the continuous phases (Fig. S18). Thus, CP formation in NIPS is more consistent with the typical nucleation-growth mode [45] rather than the spinodal mode [46] in phase separation.

The interaction energies among nonsolvents, solvents and polymers were calculated using density functional theory (DFT) (Fig. S19). As shown in Fig. 3b, there were significant differences between the interaction energies of Type I and Type II nonsolvents with DMAc. For the 15 wt.% PBI–DMAc solution, the energy changes (Δ*E*_NIPS_) in the NIPS process could be estimated by applying thermodynamic cycles (Fig. S20) to the PBI–DMAc and DMAc–nonsolvent interaction energies (*E*_PD_ and *E*_DN_, respectively). Δ*E*_NIPS_ (Table S7) for Type I nonsolvents (< −5 kcal mol^−1^) were significantly lower than those for Type II nonsolvents (>−1 kcal mol^−1^), consistent with trends in *E*_DN_.

A quantitative analysis was conducted to facilitate the rational design of membranes. The correlation between Δ*E*_NIPS_ and membrane microstructures was further clarified. Considering rapid polymer solidification and limited pore fusion, the nucleation number was approximated by the CPs number. The cross-sectional SEM images of porous membranes were utilized to quantify the area density of CPs and measure the CP size (Fig. 3c and Fig. S21). According to classical nucleation theory (Fig. S22) [47,48], higher interfacial tension rendered nucleation and growth more difficultly. The interfacial tension between the polymer-poor phase (primarily nonsolvent and DMAc) and the polymer-rich phase (primarily PBI and DMAc) could be approximately estimated by the interaction energies of nonsolvent–PBI (*E*_NP_) and nonsolvent–PBI–DMAc (*E*_NPD_). Higher *E*_NP_ and *E*_NPD_ in Type II *vs*. Type I systems (Fig. S19) indicated that Type II systems require overcoming higher interfacial energy to form CP nuclei, and thus exhibit a lower CP nucleation density than Type I systems (Fig. 3d). Subsequent slower polymer solidification allowed CPs to grow larger in Type II than in Type I systems (Fig. 3d). Therefore, the pore size was linked to interphase exchange rate and polymer solidification rate, whereas the area density was impacted more by thermodynamic factors.

Surprisingly, there was almost no correlation between Δ*E*_NIPS_ and the area density of CPs (*R*^2^ = 0.283, Fig. S23). This results from the significant deviation observed for water since this nonsolvent pulled down the overall correlation. Considering that the area density was related to the spatial geometric factors, the nonsolvent molecular volume (*V*_m_) was introduced to correct Δ*E*_NIPS_, yielding *E*_V_, as shown in Eq. 4:


(4) \begin{eqnarray*}
{{{E}}}_V = \frac{{\Delta {{{E}}}_{\rm {NIPS}}}}{{{{{V}}}_{\rm m}}}.
\end{eqnarray*}

Plotting the area density of CPs against *E*_V_ (Fig. 3e) revealed a very strong correlation (*R*^2^ = 0.969). This supports the hypothesis on the role of molecular volume, enhancing the predictive accuracy for the relationship between nonsolvents and membrane microstructure. For instance, with similar Δ*E*_NIPS_, the lower *V*_m_ of the nonsolvents leads to higher sum of |Δ*E*_NIPS_| per unit volume, thereby increasing the nucleation density of CPs. This explains why the small variations in Δ*E*_NIPS_ could cause significant disparities in CP nucleation density (such as water *vs*. EtOH, Fig. 3c and Fig. S23). By contrast, the correlation was significantly lower when the independent variable was replaced with parameters calculated via Flory-Huggins theory (*R*^2^ = 0.275) and Hansen solubility parameters (*R*^2^ = 0.624) (Fig. S24 and Table S8). This further highlights the rationality of using *E*_V_ to predict membrane microstructure.

Membrane formation kinetics in the NIPS process were analyzed by plotting the square of the moving distance of the phase separation frontier (*x*^2^) versus time (*t*) in Type I systems (Fig. 3f). Under the quasi-double infinite diffusion conditions (Fig. S25), the *x*^2^–*t* linear correlation conformed to the relevant Fick’s equation (Fig. 3f). The slope reflects the rate of phase separation, namely the membrane formation kinetics. Diffusion coefficient (*D*, Fig. S26), calculated using *δ*_N_ (Table S6), appeared to have no correlation with membrane formation kinetics superficially (Fig. 3f) because nonsolvent concentration (*C*) in the ternary system during polymer precipitation was not considered. By substituting *D* and *C*, Python-based simulations of the diffusion process and membrane formation kinetics (Figs S27–S30) matched the actual observations well (Fig. 3f). Furthermore, DMAc diffusion during the NIPS process was also studied using a custom-designed device (Fig. S31), further confirming compliance with Fick’s equation. Unlike previous reports, interference from convection and spatial inhomogeneity, caused by macrovoids, was completely eliminated for the first time, thereby proving that CPs formed via nucleation phase separation driven by molecular diffusion.

### The overall mechanism of NIPS

**Figure 4.**
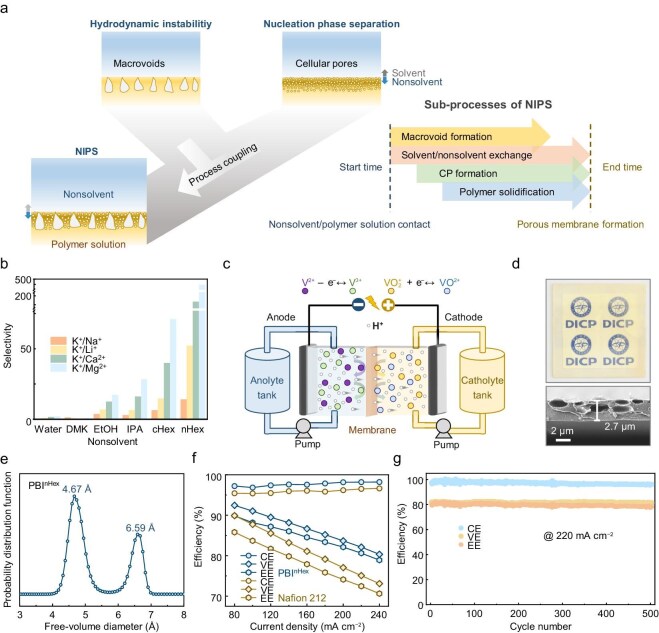
Summary and application of the NIPS mechanism. (a) Schematic diagram summarizing the NIPS mechanism. CP formation is a typical result of the nucleation-driven phase separation. Hydrodynamic instability causes macrovoid formation. The coupling of CPs and macrovoids forms the resultant porous membrane microstructures. (b) Ion selectivity of PBI membranes prepared using different nonsolvents. Membranes prepared with Type II nonsolvents present lower CP density and relatively denser pore walls, causing greater ion-sieving capacity than those with Type I nonsolvents. (c) Schematic diagram of a vanadium flow battery (VFB). The VFB uses V^2+^/V^3+^ and VO^2+^/VO_2_^+^ as the negative and positive redox-active species, respectively, and H_2_SO_4_ as the supporting electrolyte. VFBs are highly promising for large-scale energy storage. (d) Photo (top) and cross-sectional SEM image (bottom) of a PBI membrane prepared in nHex (PBI^nHex^). SEM magnification: ×7500. The semi-transparent PBI^nHex^ is only 2.7 μm thick. (e) Free-volume diameter of PBI^nHex^ obtained from PALS. (f) Efficiencies of VFBs assembled with PBI^nhex^ and Nafion 212 membranes in the current density range of 80–240 mA cm^−2^. The VFB with PBI^nHex^ always displays higher coulombic efficiency (CE) and voltage efficiency (VE), thus achieving higher energy efficiency (EE), in contrast to Nafion 212. (g) Cycling performance of the VFB assembled with PBI^nHex^ at 220 mA cm^−2^. Polymer solution: (b–g) 15 wt.% PBI–DMAc solution. Casting thickness: (b) 100 μm, (d–g) 10 μm.

As mentioned above, NIPS involves the coupling of multi-processes, mainly including solvent–nonsolvent exchange, CP and/or macrovoid formation, and polymer solidification (Fig. 4a). During the NIPS process, macrovoid appearance can obviously enhance mass transfer in the ternary system, thereby accelerating all subprocesses. This can well explain the fact that pore formation superficially correlated with membrane formation rates, as commonly called ‘instantaneous’ or ‘delayed’ phase separation [5,49]. On the contrary, a thick precipitated polymer layer only containing CPs will slow down all subprocesses, resulting in fusion of CPs to minimize the interfacial energy. Macrovoids may then start to form from these large fused CPs (Fig. S14).

### Structure–property relationship of porous membranes

Porous membranes with different structures, prepared in different nonsolvents, correspond to distinct properties. Ion separation performance is crucial for membrane processes such as energy storage batteries and lithium extraction from salt lakes. Therefore, the ion separation performance of porous membranes was measured by using an H-type device (Fig. 4b and Fig. S32) [50]. For membranes prepared in Type I nonsolvents (namely Type I membranes), although the macrovoid formation led to a decrease in selectivity, the more critical factor was that the interconnected CP structures prevented the achievement of high ion selectivity. In contrast, membranes prepared in Type II nonsolvents (namely Type II membranes) formed suitable ‘ion sieves’ based on dense polymer matrix, endowing them with high ion selectivity.

To deeply investigate the structure–property relationship, a series of free-standing, thin (< 10 μm), porous membranes were produced by varying the nonsolvents (Fig. S33) and applied in vanadium flow batteries (VFBs, Fig. 4c) that hold prospects for large-scale energy storage. From Fig. S34, Type II membranes demonstrated much lower VO^2+^ permeability than Type I membranes, which was also ∼0.1% of VO^2+^ permeability for the commercial Nafion 212 (3.5 × 10^−5^ cm^2^ h^−1^), owing to the suitable ‘ion sieves’ formed in Type II membranes. Meanwhile, all membranes exhibited lower area resistances in 3 M H_2_SO_4_ than commercial Nafion 212 (Fig. S35), indicating higher proton permeability of porous membranes. All membranes also showed preferential proton conduction (Fig. S36). As a result, Type II membranes achieved higher energy efficiency (EE) in VFBs at a current density of 80 mA cm^−2^ (Fig. S37). Among those membranes, the membrane prepared in nHex (PBI^nHex^) exhibited the lowest thickness of 2.7 μm and the highest EE (Fig. 4d and Fig. S37). Positron annihilation lifetime spectroscopy (PALS) and wide-angle X-ray scattering results revealed that the free-volume diameter formed by chain packing in the polymer matrix of PBI^nHex^ ranged from 4 Å to 7 Å (Fig. 4e and Fig. S38), which was smaller than the size of hydrated vanadium ions (>8.1 Å) [51]. The highly efficient sieving of vanadium ions and protons (4.1 Å) was thus achieved, exhibiting high selectivity. Moreover, the low thickness of PBI^nHex^ further accelerated the proton transport, thereby showing low area resistance. The VFBs assembled with PBI^nHex^ always exhibited higher efficiencies than those using Nafion 212 at a current density ranging from 80 to 240 mA cm^−2^ (Fig. 4f). In particular, this VFB, assembled with PBI^nHex^, exhibited a high EE of 80.6% at 220 mA cm^−2^ and ran stably for more than 500 cycles, demonstrating high reliability (Fig. 4g). As a result, membrane microstructures can be rationally designed to achieve optimized performance.

## CONCLUSION

The formation processes of macrovoids and CPs were decoupled through the design of a fluid device to observe the NIPS process *in-situ*. Correlating macrovoid formation with the viscous fingering theory proved its regulation via hydrodynamic instability. By inhibiting macrovoid formation, it was further illustrated that CP formation was based on the nucleation-growth mechanism. To categorize nonsolvents, *E*_V_, combined with Δ*E*_NIPS_ and nonsolvent molecular volume, was defined as a more accurate thermodynamic descriptor. Nonsolvents with low *E*_V_ allowed the design of porous membranes of disconnected CPs (i.e. dense polymer matrix) in the absence of macrovoids. The prepared membranes were permeable, selective and stable, thus significantly enhancing the performance of electrochemical devices, such as VFBs. This work sheds new light on the classical knowledge of the NIPS mechanism and has proved useful to accurately tune the membrane microstructure and separation performance, which has been a long-standing challenge.

## METHODS

The experimental details can be found in the online supplementary data.

## Supplementary Material

nwag306_Supplemental_Files
