# Supplementary material for: Extending the theory of classical nonsolvent-induced phase separation to regulate membrane pores
Source: Natl Sci Rev. 2026 May 27;13(13):nwag306. doi: 10.1093/nsr/nwag306 (PMC13335106; doi:10.1093/nsr/nwag306)
Supplement: nwag306_Supplemental_Files [file nwag306_supplemental_files.zip › Supplementary data.pdf]

SUPPLEMENTARY INFORMATION

Extending the Theory of Classical Nonsolvent Induced Phase Separation to Regulate Membrane Pores

Chaoyang Jia[1,2], Chenkai Mu[1,2], Yiwen Chen[3], Hongjun Zhang[3], Willem Verfaillie[4], Scout Caspers[4], Ivo F.J. Vankelecom[4], Wenjing Lu[1*], Xianfeng Li[1*]

1Dalian Institute of Chemical Physics, Chinese Academy of Sciences, Dalian 116023, China.

2University of Chinese Academy of Sciences, Beijing 100049, China.

3State Key Laboratory of Particle Detection and Electronics, University of Science and Technology of China, Hefei 230026, China.

4Membrane Technology Group, Centre for Membrane Separations, Adsorption, Catalysis and Spectroscopy for Sustainable Solutions (cMACS), Faculty of Bioscience Engineering, KU Leuven, Leuven 3001, Belgium

***Corresponding author**: E-mails: luwenjing@dicp.ac.cn; lixianfeng@dicp.ac.cn

Supplementary Text, Figures. 1−38, Tables 1−8, Movies 1−6 and References 1−69.

TABLE OF CONTENTS

Supplementary Text……………………………………………………………………………….3
Methods and Materials……………………………………………………………………………5
Supplementary Figures……………………………………………………………..……….10
Supplementary Tables……………………………………………………………………………50
Supplementary References…………………………………………………………………….58

SUPPLEMENTARY TEXT

A brief review of phase separation approaches

Phase separation approaches primarily encompass three types: nonsolvent-induced phase separation (NIPS) [1], vapor-induced phase separation (VIPS) [2], and thermally induced phase separation (TIPS) [3]. Their fundamental distinction lies in the driving forces for phase separation: TIPS is driven by heat exchange, while NIPS is driven by matter exchange; VIPS involves both matter and heat exchange. Thus, TIPS aligns most closely with the classical thermodynamic models and can be explained well using the phase diagram [4]. In contrast, the other two methods exhibit greater complexity due to matter exchange. Among these, NIPS represents the most complex process, characterized by rapid mutual exchange between nonsolvent and solvent, accompanied by the formation of complex microstructures [5].

Current research continues to employ the binary concepts of ‘nstantaneous phase separation’ and ‘delayed phase separation’ to explain the formation of finger-like macrovoids (Mvs) and the spongy structure consisting of cellular pores (CPs) in NIPS [5−7]. However, it is crucial to clarify that the relationship between Mvs and CPs does not conform to this binary opposition; rather, Mvs manifest as a concomitant structure of CPs (**Fig. 1a, b, and Fig.S2**). Notably, CPs are universally present across all the aforementioned phase separation approaches (Table S1–S3), highlighting that CPs represent an intrinsic structure regulated by thermodynamics. Therefore, understanding CPs formation necessitates accounting for thermodynamic model. However, current investigations into CP formation remain merely at the qualitative stage.

On the other hand, research on Mvs primarily emphasized microscopic observations [8,9] and computational simulations [10,11]. Several studies have directly documented Mvs growth dynamics and morphological characteristics through microscopy techniques [8,9]. These works propose that ‘Increasing polymer viscosity’ or ‘constructing a surface skin layer’ can inhibit Mvs growth [8], and suggest their formation relates to the convection mechanism [12,13]. Additional studies have further corroborated the connection between Mvs formation and convection mechanism through computational simulations [10,11]. Nevertheless, computational simulations often rely on phenomenological equations, and the parameters governing simulation outcomes are typically based on empirical values rather than first principles. Furthermore, the temporal and spatial scales of these simulations often do not match those of actual pore formation. In NIPS systems, direct experimental validation supporting the hydrodynamic origin of Mvs remains elusive, preventing consensus regarding their formation mechanism.

Conventional theoretical frameworks, including Flory-Huggins theory [14] and Hansen solubility parameters [15], provide only approximate prediction of Mvs presence. These thermodynamic approaches prove insufficient and potentially misleading, particularly if Mvs formation follows the hydrodynamic principles. Concurrently, some studies have monitored NIPS processes using *in-situ* spectroscopic techniques (e.g., ultrasound, X-rays) and established phase separation and mass transfer kinetic models [16–20]. However, limited by the challenge of achieving simultaneous high temporal and spatial resolution, the acquired signals typically represent integrated information of multiple coupled processes. Furthermore, the integrated information cannot be directly linked to the evolution of membrane microstructures (e.g., CPs, Mvs).

In summary, despite substantial research progress, three fundamental scientific questions pertaining to NIPS require resolution: I. The formation mechanism of Mvs and corresponding

direct experimental verification; II. The thermodynamic principles underlying CP formation; III. The optimal descriptors for membrane microstructures and phase separation driving forces, along with quantitative models based on these descriptors.

METHODS AND MATERIALS

Materials

Polybenzimidazole (PBI) was synthesized according to our previous report [21]. Poly (ether sulfone) (PES) was provided by Changchun Jida Special Plastics Engineering Research Co., Ltd. N, N-dimethylacetamide (DMAc), toluidine blue (TB) and polyethylene glycol 200 (PEG 200) of analytically pure were purchased from Aladdin. Ethanol (EtOH), isopropanol (IPA), dimethyl ketone (DMK), cyclohexane (cHex) and n-hexane (nHex) of analytically pure were purchased from Kermel. All the reagents were used as received. Copper (Cu) foil of 25 μm and 50 μm thickness were purchased from Solarbio.

Preparation of polymer solution

A certain mass of PBI were dissolved in DMAc. The polymer was fully dissolved via mechanical stirring for at least 10 hours, after which the solution was allowed to stand for at least 10 hours to remove bubbles. A PES-DMAc solution was prepared using the same procedure.

Membrane preparation by nonsolvent-induced phase separation (NIPS)

A clean glass plate was used as the substrate. The polymer solution was poured onto the glass plate and cast into a liquid film using a scraper with a set thickness of 10 μm or 100 μm. The glass plate was then rapidly immersed in a nonsolvent (e.g., water, EtOH, DMK, IPA, nHex, or cHex) to induce phase separation until the membrane fully solidified. Solidified membranes were stored in deionized water for subsequent use.

Membrane preparation by vapor-induced phase separation (VIPS)

A clean glass plate was used as the substrate. The polymer solution was poured onto the glass plate and cast into a liquid film using a scraper with a set thickness of 100 μm. The glass plate was then rapidly exposed into nonsolvent vapor in a chamber maintained at 25 °C with 100% relative humidity for 4 hours until the membrane fully solidified. Solidified membranes were stored in deionized water for subsequent use.

Membrane characterization

Cross-sectional morphologies of the membranes were observed using a scanning electron microscope (SEM, JSM-7800F) with an acceleration voltage of 3 kV and a focused ion beam-SEM (FIB-SEM, FEI Strata 400 S and Thermo Scientific Apreo 2C) with an acceleration voltage of 5 kV. For cross-sectional structure analysis (SEM), membrane samples were frozen and fractured in liquid nitrogen. Sample cross-section observed by the FIB-SEM was obtained by FIB etching. All samples were sputter-coated with Pt or C before observation. Wide-angle X-ray scattering (WAXS) instrument (Xeuss 2.0, Xenocs) exhibited a minimum scattering vector Q_{min} of approximately 0.817 nm^{-1}. When the direct beam was positioned at the center of the two-dimensional (2D) pattern, the maximum scattering vector Q_{max} was approximately 18 nm^{-1} (with Q_x ranging from −10.8 nm^{-1} to 11.3 nm^{-1} and Q_z ranging from −12.5 nm^{-1} to 16.0 nm^{-1}). When the direct beam was located at the bottom-left corner of the 2D pattern, the Q_{max} increased to approximately 26 nm^{-1} (with Q_x ranging from −3.1 nm^{-1} to 18.1 nm^{-1} and Q_z ranging from −3.0 nm^{-1} to 23.4 nm^{-1}). WAXS samples were prepared by stacking multiple membranes.

Positron annihilation lifetime spectroscopy (PALS)

Owing to the challenges in satisfying the conventional PALS spectrometer sample requirement (total thickness exceeding 2 mm), PALS measurements were performed using a custom-designed

spectrometer equipped with a 200 ps time resolution and a 30 μCi (1.11 MBq) ^{22}Na radioactive source [22,23]. Membrane sample was folded to attain a thickness of approximately 0.1 mm for measurement, with over 4 million counts accumulated for a positron lifetime spectrum.

The continuous positron lifetime distribution was evaluated using the MELT procedure [24], an approach rooted in the maximum entropy principle that obviates the need for standard spectra calibration.

NIPS observation cell (OC)

Two clean glass slides with uniform thickness were employed. On the bottom slide, two parallel lines were marked along the long edge at approximately 1/7 (Line I) and 3/7 (Line II) of the total length (**Fig. S5**). Line I served as the alignment reference for positioning the top slide, while Line II delineated the outer boundary for gasket placement. A central crosshair (Mark III) was designated as the dropping position for the polymer solution. Standard 25-μm-thick or 50-μm-thick Cu foils were used as gaskets, with three pieces placed symmetrically around Mark III, aligned with Line II. Following dropping of the polymer solution onto Mark III, the top slide was positioned over the bottom slide using Line I as a guide, leaving approximately 1/7 of the bottom slide uncovered. The assembly was secured by clamping all four corners with spring clips.

Microscope observation

Following completion of the OC assembly and microscope focusing, video recording began. A small volume of nonsolvent was introduced to the uncovered region of the bottom glass slide. Capillary action drew the nonsolvent into the slit between the two slides, where it contacted the polymer solution and induced phase separation. *In-situ* observations were performed using an Andor Live Cell Station (Revolution WD) equipped with a 405 nm laser and an optical microscope (CM30W-HK810). *Ex-situ* analyses were conducted using a two-photon confocal microscope system (FV1000MPE) with excitation wavelengths of 405 nm and 488 nm.

Saturation solubility and cloudy point titration

In the saturation solubility titration, 2.5 g of DMAc was placed in a glass bottle and magnetically stirred. Nonsolvent was added dropwise under continuous stirring until the solution became turbid and no visible changes were observed for over 10 minutes. For the cloudy point titration, the procedure was modified by replacing DMAc with a 15 wt.% PBI-DMAc solution. The cloud point was defined as the point at which polymer particles precipitated, with no significant change persisting for more than 10 minutes.

Counting Cellular pores (CPs) density

Using cross-sectional SEM images of porous membranes prepared with different nonsolvents, a square region was demarcated to encompass the maximum number of CPs. The number and area of pores were quantified using the open-source software Image J [25,26]. The procedure was as follows: SEM images containing CPs were imported into ImageJ and converted to 8-bit grayscale format (Image → Type → 8-bit), and the image scale was calibrated (Analyze → Set Scale). CP regions were then highlighted by threshold adjustment (Image → Adjust → Threshold), and interconnected CPs were separated using the watershed algorithm (Process → Binary → Watershed). Finally, noise particles smaller than 1/40 of the average CP size were excluded, and statistical data was obtained via particle analysis (Analyze → Analyze Particles). The statistics included the total number, total area, and area fraction of CPs, as well as the cross-sectional area of individual CP. The equivalent circular diameter (d_c) of each CP was calculated from the CP area (A) as follows:

$$d_c = 2\sqrt{\frac{A}{\pi}} \tag{S1}$$

Ion selectivity

An H-type glass device (**Fig. S32**) was used to measure the ion selectivity of membranes. A hydrophilic carbon paper electrode and an L-shaped salt bridge were then inserted into each half cell, with Ag/AgCl reference electrodes placed within each salt bridge. The membrane was then fixed between half cells of this device. Equal volumes of identical metal salt solutions were subsequently injected into both half cells. Current–voltage (I–V) curves of the H-type device with and without the membrane were recorded using a Series 2600B SourceMeter® System. Prior to testing, the membranes were pre-immersed in the corresponding metal salt solution for at least 6 hours to ensure complete pore infiltration. Among the metal salts, the concentration of KCl, NaCl, LiCl were 0.5 M, while the concentration of $CaCl_2$ and $MgCl_2$ were 0.25 M, ensuring an identical Cl^- concentration across all solutions. Since Cl^- transported across the membrane more readily than cations, it was necessary to maintain consistent Cl^- concentrations to reflect differences in cation transport abilities.

The selectivity factor (*S*) was calculated using the following equation:

$$S_{12} = \frac{\left(R_{M2} - R_{E2}\right) Z_1}{\left(R_{M1} - R_{E1}\right) Z_2} \tag{S2}$$

Where, R_{M1} and R_{E1} are the resistance of H-type device with and without a membrane in solution 1 according to the I–V curve slope, respectively. R_{M2} and R_{E2} are the corresponding resistance in solution 2 according to the I–V curve slope, respectively. Z_1 and Z_2 represent the charge number of the cations in solution 1 and 2, respectively.

Area resistance (AR)

The AR of the membrane was also measured using an H-type glass device. Both half cells were filled with 3 M H_2SO_4. I–V curves of the device with and without the membrane were recorded using a Series 2600B SourceMeter® System. The resistance derived from the slope of the obtained I–V curve. The AR of the membrane was calculated using the following equation.

$$\text{AR} = R_M - R_E \quad A_M \tag{S3}$$

Where, R_M and R_E are the device resistance with and without a membrane, respectively. A_M is the effective area of the membrane.

Ion transport number

The two half cells of the H-type glass device were respectively filled with 0.3 M and 0.1 M H_2SO_4, with all subsequent procedures consistent with the AR test protocol. The Donnan potential (*E,* unit: mV) was obtained as the *V* value at *I*=0 from the acquired I–V curve. The transport numbers of cation (t^+) and anion (t^-) were calculated using the following equation:

$$E = -59 \ln\left(\frac{0.3}{0.1}\right)\left(t^+ - t^-\right) \tag{S4}$$

$$t^+ + t^- = 1 \tag{S5}$$

VO^{2+} permeability

The VO^{2+} permeability of the membrane was evaluated using a traditional permeation device. The two half cells of device were separated by a 3 cm×3 cm membrane. 84 mL of 1.5 M $VOSO_4$ + 3.0 M H_2SO_4 was added to the left cell, and 84 mL of 1.5 M $MgSO_4$ + 3.0 M H_2SO_4 was added to the right cell. 1.5 M $MgSO_4$ was used to balance the osmotic pressure of 1.5 M $VOSO_4$. The solutions in both cells were stirred continuously to weaken concentration polarization. After a fixed time interval, 3 mL of sample solution was taken out from the right cell, and 3 mL of fresh original solution (1.5 M $MgSO_4$ + 3.0 M H_2SO_4) was added to maintain volume consistency. The VO^{2+} concentration in the sample solution was measured by a UV-Vis spectroscopy (UV-2600, Shimadzu). The VO^{2+} permeability (P) was calculated using the following equation:

$$V_R \frac{dC_R(t)}{dt} = A\frac{P}{L}\left(C_L - C_R(t)\right) \quad \text{(S6)}$$

Where, V_R and $C_R(t)$ is volume and VO^{2+} concentration of the right cell, A membrane area, t time, C_L the VO^{2+} concentration of the left cell. Since $C_R(t) << C_L$, $C_R(t)$ to the right of the equation in Eq. S6 can be ignored.

DMAc mass transfer

A test tube of fixed length and radius was filled with 15 wt.% PBI–DMAc solution. The tube was secured onto the inner wall of a 250 mL beaker, after which the 200 mL nonsolvent was quickly poured into the beaker. Magnetic stirring was immediately initiated while starting the timer. Every 15 minutes, 3.5 mL of the nonsolvent sample was withdrawn from the beaker and replenished with an equal volume of pure nonsolvent. After five sample was collected, the concentration of DMAc in the samples was determined using UV-Vis spectroscopy (UV-2600, Shimadzu).

Vanadium flow battery (VFB) performance

The VFB single cells were assembled with graphite plates, carbon felt electrodes (effective area: 6 cm ×8 cm and thickness: 5 mm) and a membrane (effective area: 6 cm ×8 cm). The catholyte was 60 mL of 1.5 M VO^{2+}/VO_2^+ + 3 M H_2SO_4 and the anolyte was 60 mL of 1.5 M V^{2+}/V^{3+} + 3 M H_2SO_4. The electrolytes were pumped from the electrolyte storage tanks into the cell cavity driven by the magnetic drive pumps. ArBin BT2000 was used to perform galvanostatic charge and discharge tests on VFB single cells. The cut-off voltages were set to 1.55 V and 1.0 V, respectively. The operating current density range was 80-240 mA cm^{-2}.

Binding energy calculation

All calculations were performed using density functional theory (DFT) with the Gaussian 16 package [27]. Geometry optimizations were performed using the M06-2X [28] density functional in conjunction with the def2-TZVP basis set [29]. Grimme's D3 dispersion correction [30] was included to account for van der Waals interactions. Frequency calculations were performed at the same level of theory to confirm that the optimized structures corresponded to minima on the potential energy surface and to obtain zero-point vibrational energies and thermal corrections. All calculations were performed with the ultrafine integration grid. The binding energies (ΔE) were evaluated as the following equation:

$$\Delta E = E(\text{complex}) - \left[E(\text{A}) + E(\text{B})\right] \quad \text{(S7)}$$

Where E(complex) represents the total electronic energy of the optimized complex, and E(A) and E(B) are the energies of the isolated components. The counterpoise procedure was applied to correct for basis set superposition error.

Code availability

The source code for the model is available via GitHub at https://github.com/jcy-DICP/Diffusion-simulation.

SUPPLEMENTARY FIGURES

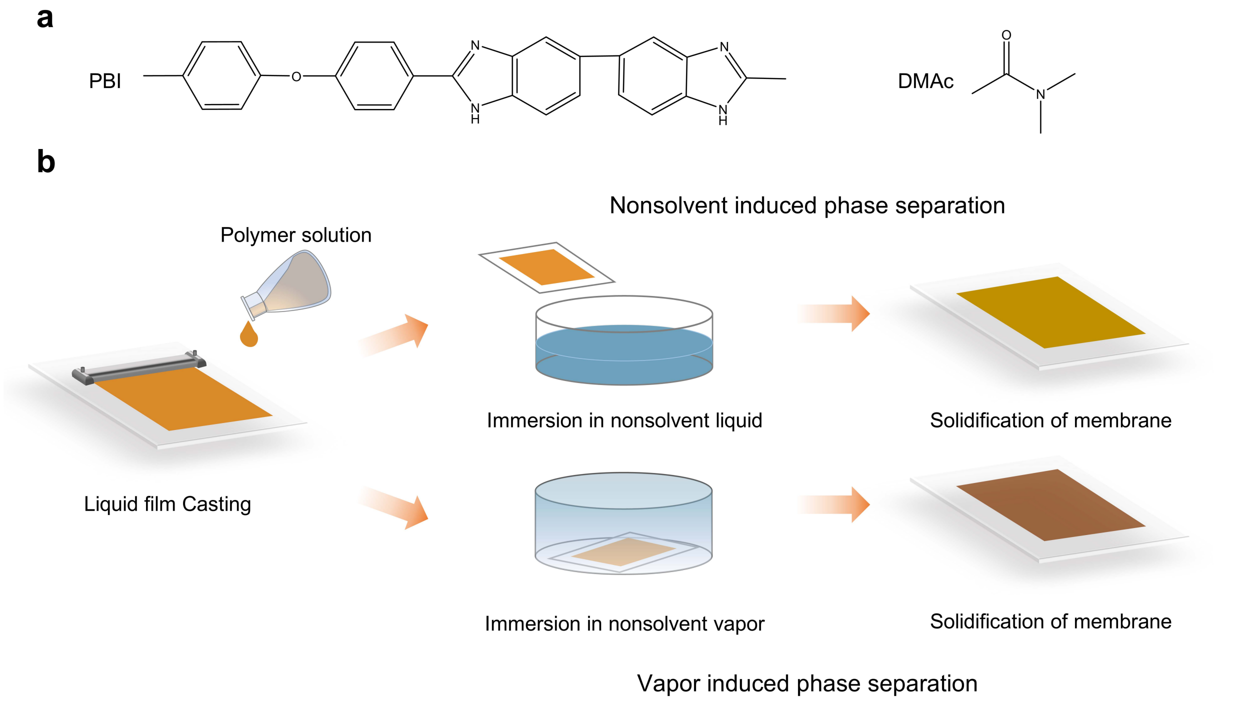

Fig. S1. (a) Chemical structures of PBI and DMAc. (b) Schematic illustrations of porous membrane fabrication via NIPS and VIPS methods.

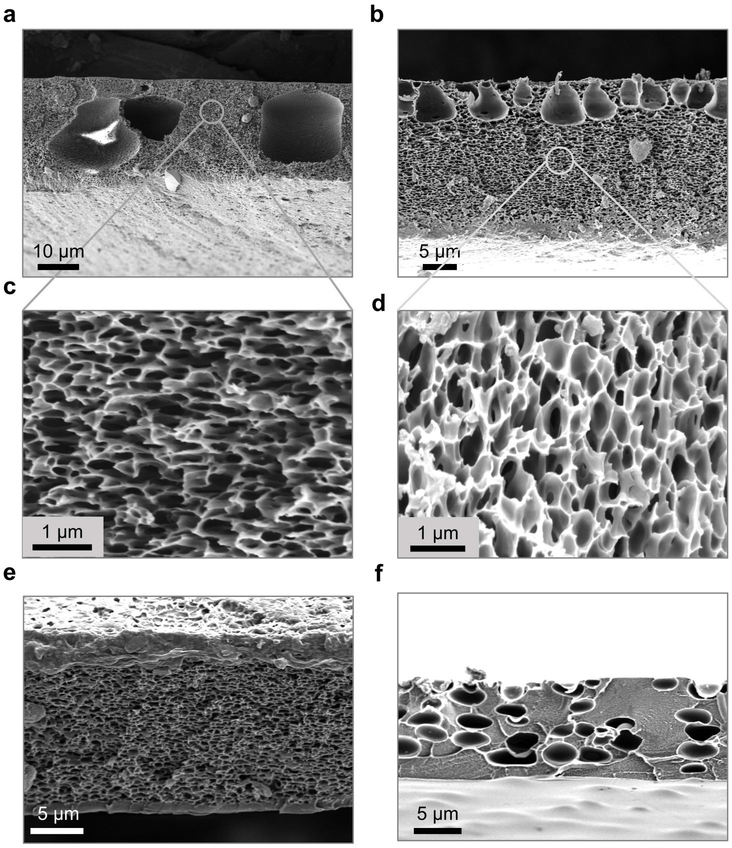

Fig. S2. Cross-sectional SEM images of porous membranes prepared via the NIPS method using different nonsolvents: (a, b) Membranes with Mvs surrounded by CPs. (c, d) Details of the CPs surrounding Mvs. (E, F) Membranes composed solely of CPs. Nonsolvent: (a, c) DMK, (b, d) EtOH, (e) IPA, and (f) cHex. Polymer solution: 15 wt. % PBI–DMAc solution. Casting thickness: 100 μm. SEM magnification: (a) ×1500; (b) ×2500; (c) ×10000; (d) ×20000; (e) ×2500; (f) × 2500. Mvs and CPs exhibit completely distinct characteristics: Mvs display a side-by-side growth morphology with sizes gradually increasing from the top to the bottom of the membrane, presenting pear-shaped or finger-like features; in contrast, CPs share a common characteristic in that they are of being ellipsoidal in shape and distributed throughout the entire membrane cross-section. **Figure S17** shows that the polymer matrix (namely pore walls) of the membrane fabricated in nHex is relatively dense, with no small micropores present. Furthermore, microscopic observations in **Fig. S18** reveal that CPs prepared in various nonsolvents all exhibit a circular morphology and differ only in their packing arrangement. Accordingly, it is fully reasonable to classify the membrane cross-sectional structures in **Figs. 1a, 1b and S2** into Mvs and CPs. Furthermore, closely packed CPs are formed in water, EtOH, IPA and DMK, whereas dispersed CPs are generated in cHex and nHex.

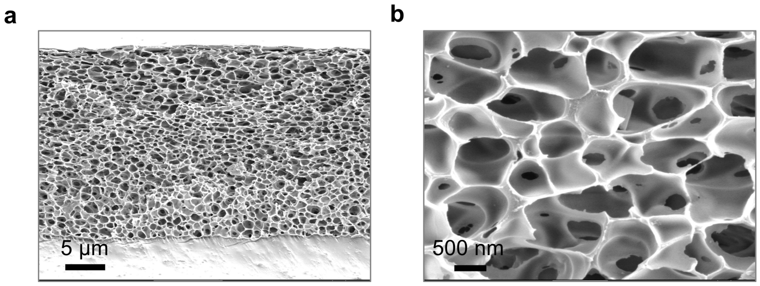

Fig. S3. (a) Cross-sectional SEM image of a porous membrane only with CPs prepared by the VIPS approach. (b) The enlarged SEM image of A. Polymer solution: 15 wt.% PBI–DMAc solution. Nonsolvent vapor: water vapor. SEM magnification: (a) ×2500; (b) ×20000;

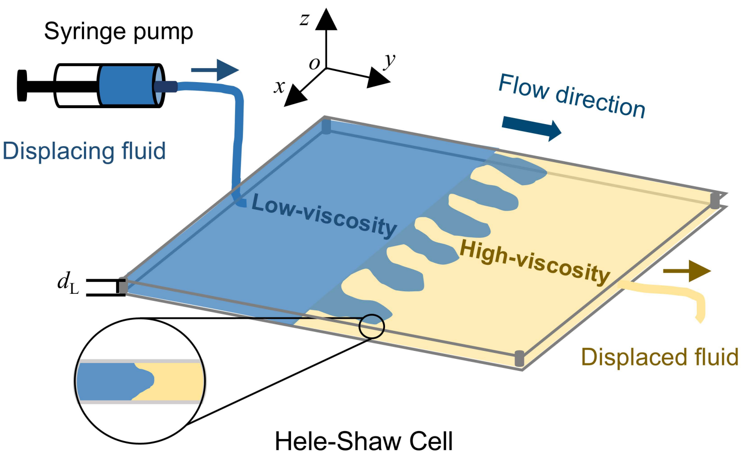

Fig. S4. The viscous fingering pattern in the Hele-Shaw cell [31–33]**.** In the Hele-Shaw cell, when a low-viscosity liquid displaces a high-viscosity liquid, a finger-like interface forms at the fluid front. From **Figs. S2–S4**, the finger-like Mvs and the viscous fingering pattern are highly similar. Therefore, we assume that the Mv formation involves the hydrodynamic factors.

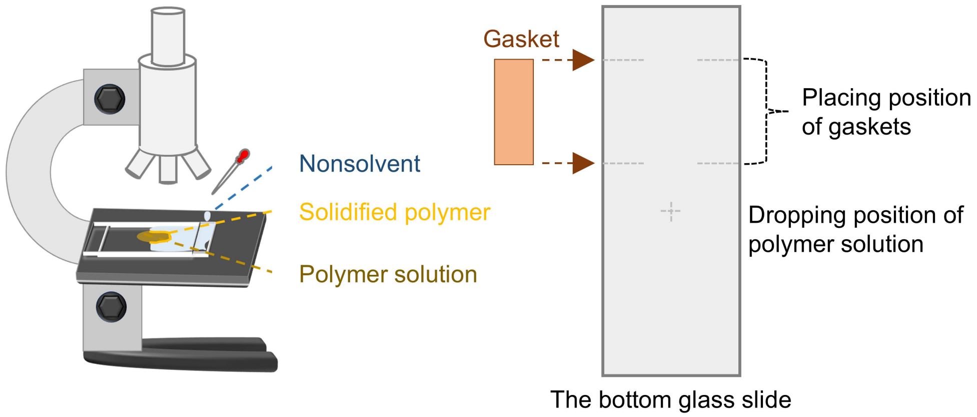

Fig. S5. Schematic diagram of the microscope observation device and the installation details of OC. According to the classical Lucas-Washburn (L-W) model [34], the capillary transport equation is expressed as:

$$F_\gamma - F_f = \frac{2\gamma\cos\theta}{R} - \frac{8\mu L\frac{dL}{dt}}{R^2} = 0 \tag{S8}$$

Here, F_γ is capillary driving force F_f viscous resistive force. γ interfacial tension. θ contact angle, R curvature radius of the capillary tube, L liquid displacement distance, and t time.

Thus, it can be concluded that both F_γ and F_f decrease with increasing R, but F_f decays significantly faster due to its $1/R^2$ dependence. Through force balance and integration, the L-W equation is derived as follows:

$$L^2 = \frac{\gamma R\cos\theta}{2\mu}t \tag{S9}$$

By further differentiation, the liquid velocity can be derived as:

$$v = \sqrt{\frac{\gamma R\cos\theta}{8\mu}\frac{1}{t}} \tag{S10}$$

As indicated by Equation Eq. S10, the nonsolvent velocity increases with the increase in slit width ($\approx R$).

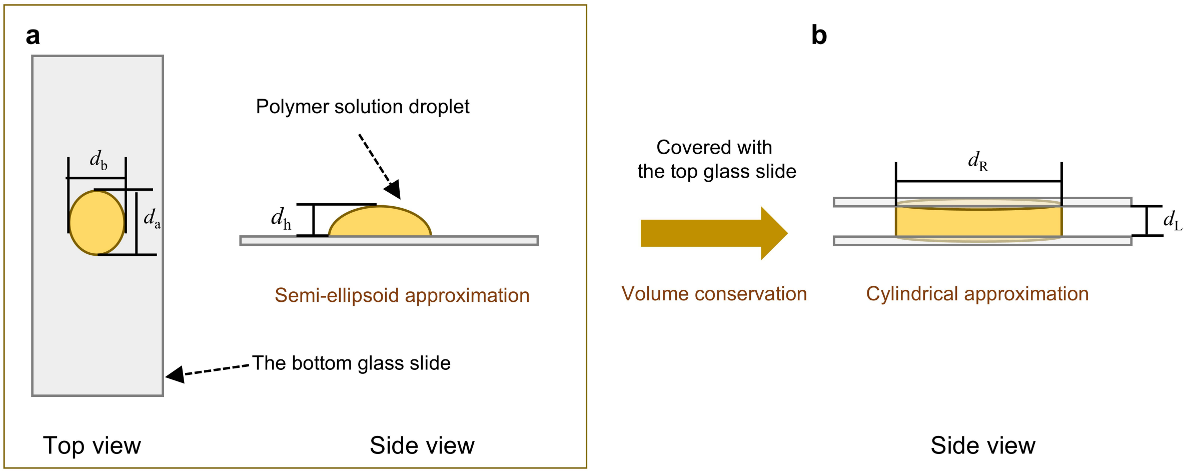

Fig. S6. Schematic diagram for estimating the slit width formed by two glass slides in the OC without gaskets. (a) Schematic diagram of a polymer solution droplet on a clean glass slide. (b) Schematic diagram a polymer solution droplet after covering the top glass slide. d_a and d_b are the major and minor axes of the approximately elliptical droplet, respectively. d_h is the vertical height of the droplet. d_R and d_L are the bottom diameter and vertical height (e.g. slit width) of the cylindrical droplet held by the glass slides, respectively.

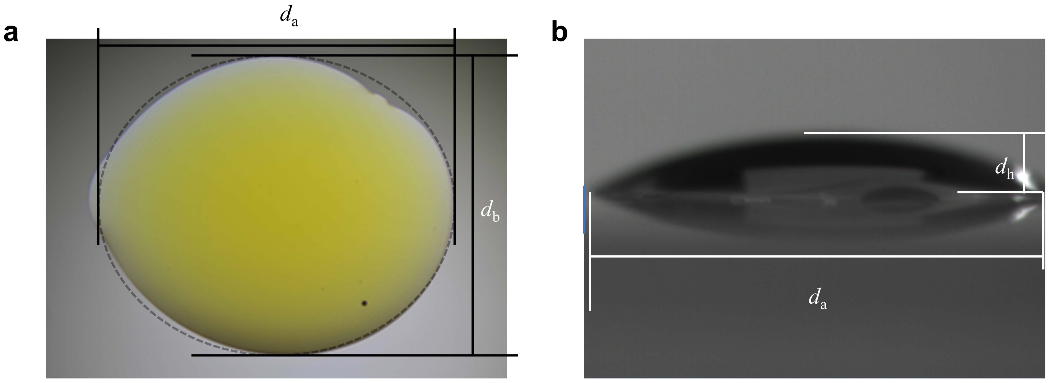

Fig. S7. (a) Top view and (b) side view of a polymer solution droplet. Due to solvent evaporation and air humidity, top and side views are not easily obtainable with limited exposure time. Therefore, the relationship between d_h and d_a can be estimated quickly. d_h was approximately equal to 1/10 of d_a, a relationship affected by gravity and interfacial tension.

According to **Figs. S6 and S7**, d_L can be estimated as the slit width using the following equation:

$$d_L = \frac{\frac{1}{2} \times \frac{4}{3} \pi (\frac{d_a}{2} \frac{d_b}{2} d_h)}{\pi (\frac{d_R}{2})^2} = \frac{d_a^2 d_b}{15 d_R^2} \tag{S11}$$

The results were listed in Table S5, when the gaskets were removed, the slit width was about 10 μm.

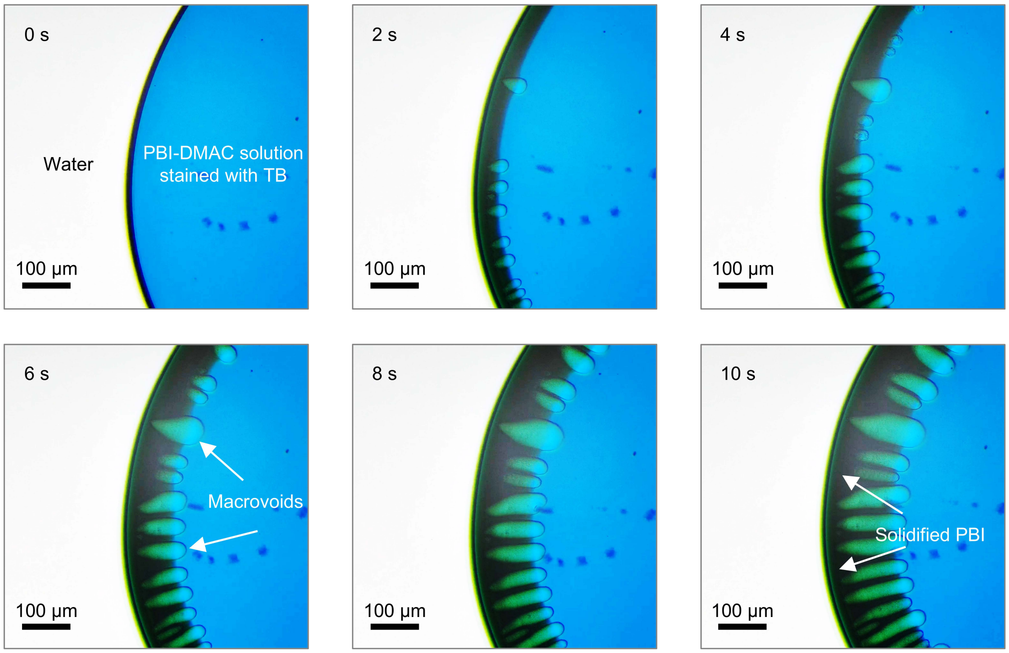

Fig. S8. Microscopic observation of the Mv formation in water-induced phase separation of TB-stained polymer solution (15 wt.% PBI–DMAc). OC: 50 μm gaskets. Since TB is easily soluble in DMAc and water, it can be used to stain the polymer solution to detect the solvent/nonsolvent content in Mvs. **Figure S8** shows that the color in Mvs was lighter, indicating that nonsolvent entered the Mvs in a short time. As a result, it was the fluid behavior rather than diffusion behavior that can better explain the Mv formation.

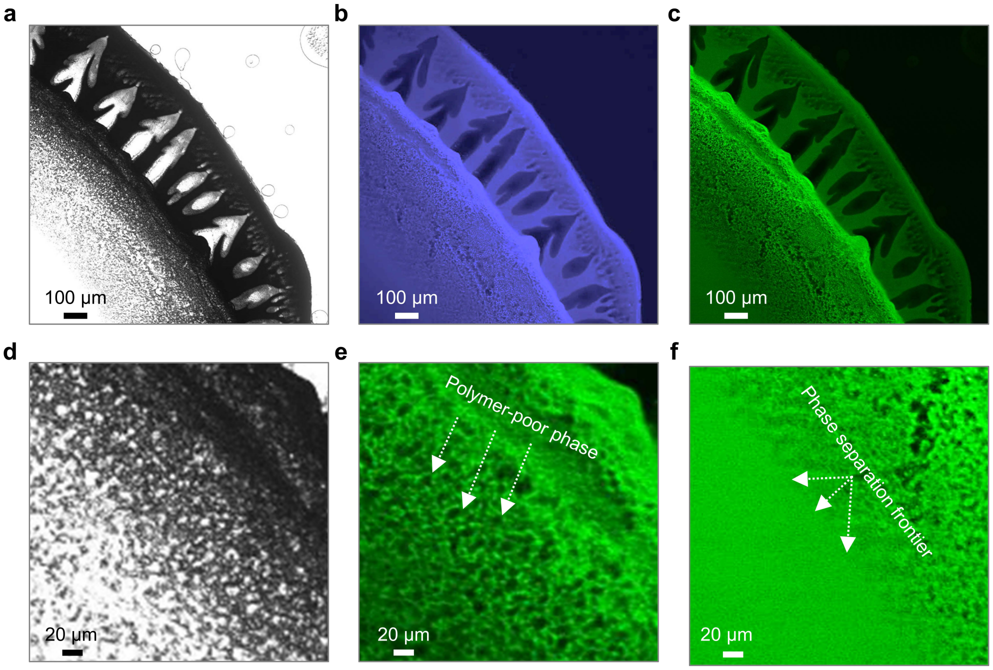

Fig. S9. Fluorescence microscopic images of (a–c) Mvs, (d–e) CPs, and (f) the phase separation frontier. Polymer solution: 15 wt.% PBI–DMAc solution. Nonsolvent: water. OC: 50 μm gaskets. The polymer-poor phase (the black spot) indicated by the arrow in **Fig. S9e** was the CPs precursor in a mixture mainly composed of nonsolvent and solvent. The phase separation frontier was the boundary between phase-separated region and phase-unseparated region.

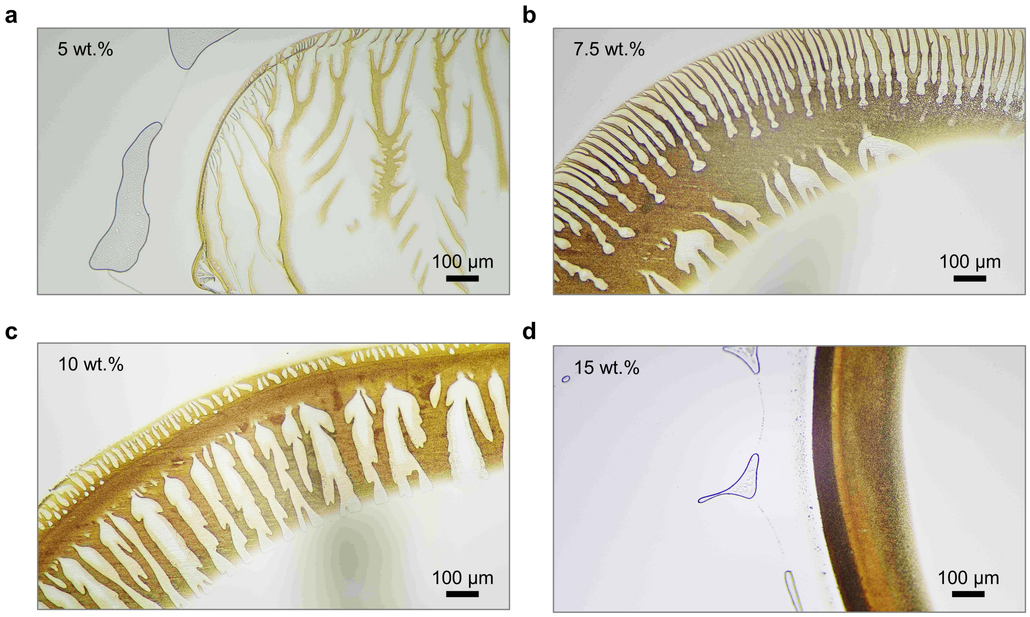

Fig. S10. Microscopic structures formed in process of NIPS with different concentration of polymer solutions. (a) 5 wt.%, (b) 7.5 wt.%, (c) 10 wt.% and (d) 15 wt.% PBI–DMAc solution. Nonsolvent: water. OC: no gaskets. The lower concentration of the polymer solution caused lower solution viscosity, resulting in more Mvs. When the concentration of the polymer solution was as high as 15 wt.%, no Mvs could be found. That phenomenon indicated that the increase in the polymer viscosity could buffer the impact of nonsolvent, leading to the more stable interface to inhibit the Mv formation.

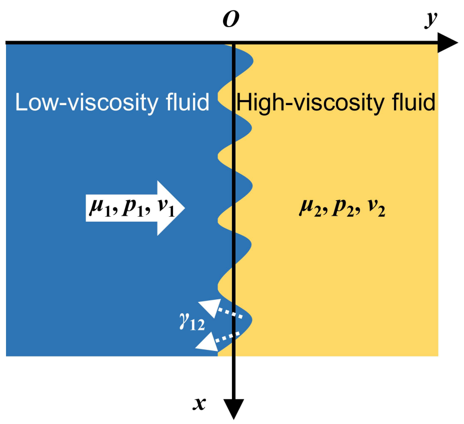

Fig. S11. Schematic diagram of interface-wave in a rectangular coordinate system. Of note, the 'low-viscosity' and 'high-viscosity' specifically compare the viscosity of fluid 1 and fluid 2**.** The originally proposed mathematical model for Saffman-Tayor instability is based on the interface-wave assumption [35,36]. Given a fixed stationary coordinate system (**Fig. S11**), a low-viscosity fluid 1 (viscosity: μ_1, velocity: v_1, pressure: p_1) displaces the high-viscosity fluid 2 (viscosity: μ_2, velocity: v_2, pressure: p_2) and induces multi-finger-like patterns at the interface. Assuming that the displacement velocity is constant, namely $v= v_1= v_2,$ the two-phase interface disturbance wave is set to $\hat{\eta}\,(x,\ t)$ (position: x, time: t), and the position expression (y) of the phase interface in the stationary coordinate is as follows:

$$y = vt + \hat{\eta}(x,t) = vt + |\,\hat{\eta}\,|\, e^{\sigma t}\sin(kx+\theta) \tag{S12}$$

Where, $|\hat{\eta}|$, σ, k and θ are the amplitude, amplification rate, wave-vector and phase of wave, respectively.

According to Darcy's law, the pressure on both sides of the phase interface is as follows:

$$\nabla p_{\mathrm{j}} = -\frac{12\mu_{\mathrm{j}}}{d_{\mathrm{L}}^{2}} v_{\mathrm{j}} \quad \text{j=1, 2} \tag{S13}$$

Where, d_L is slit width formed by the two glass slides.

Since fluid is incompressibility, $\nabla \bullet \langle v \rangle = 0$ and pressure meets the follow equation:

$$\nabla^2 p = 0 \tag{S14}$$

The total pressure fields with disturbance added can be written as follows:

$$p_{\mathrm{j}} = p_{\mathrm{j}}^{0} + \hat{p}_{\mathrm{j}} \quad \text{j=1, 2} \tag{S15}$$

By integrating Eq. S13 and setting the pressure to be 0 on the interface located at $y=vt$, we can get:

$$p_{\mathrm{j}}^{0} = \frac{12\mu_{\mathrm{j}}}{d_{\mathrm{L}}^{2}} v(y - vt) \quad \text{j=1, 2} \tag{S16}$$

According to Eq. S12, S14 and S15, the general solution of perturbation pressure can be:

$$\hat{p}_1 = A_1 e^{\sigma t + ky}\sin(kx+\theta) \tag{S17}$$

$$\hat{p}_2 = A_2 e^{\sigma t - ky}\sin(kx+\theta) \tag{S18}$$

According to the continuity of velocity on the interface, namely boundary conditions, using Eq. S13 and keeping only the first-order terms by taking $y=vt$, we thus can determine A_1 and A_2 as follows:

$$\frac{d_{\mathrm{L}}^{2}}{12\mu_{\mathrm{j}}}\left(\frac{\partial p_{\mathrm{j}}}{\partial y}\Big|_{y=vt}\right)=\frac{\partial y}{\partial t}=v+\sigma\hat{\eta}\qquad \mathrm{j}=1,\,2 \tag{S19}$$

Hence,

$$A_{1}=\frac{12\mu_{1}\sigma\,|\,\hat{\eta}\,|}{kd_{\mathrm{L}}^{2}}e^{-kvt} \tag{S20}$$

$$A_{2}=-\frac{12\mu_{2}\sigma\,|\,\hat{\eta}\,|}{kd_{\mathrm{L}}^{2}}e^{kvt} \tag{S21}$$

The pressure is also continuous at $y=vt+\hat{\eta}$. Considering the additional pressure (p_{γ}) caused by interfacial tension, we can get:

$$p_{2}-p_{1}=p_{2}^{0}-p_{1}^{0}+\hat{p}_{2}-\hat{p}_{1}=p_{\gamma} \tag{S22}$$

For the last two terms, we take the first-order term of y (i.e. $y = vt$) and substitute it into Eq. S17, S18 and S20 to S22 to obtain:

$$\frac{12\mu_{2}}{d_{\mathrm{L}}^{2}}v\hat{\eta}-\frac{12\mu_{1}}{d_{\mathrm{L}}^{2}}v\hat{\eta}-\frac{12\mu_{2}\sigma}{kd_{\mathrm{L}}^{2}}\hat{\eta}-\frac{12\mu_{1}\sigma}{kd_{\mathrm{L}}^{2}}\hat{\eta}=p_{\gamma} \tag{S23}$$

The additional pressure due to interfacial tension is:

$$p_{\gamma}=\frac{\gamma}{R} \tag{S24}$$

Where, γ is the interfacial tension and R is the interfacial curvature radius.

Because $\hat{\eta}$ is a small perturbation wave, $\ddot{\eta}$ can be approximately used to calculate the radius of curvature, so:

$$\frac{1}{R}=\frac{\ddot{\eta}}{(1+\dot{\eta})^{\frac{3}{2}}}\approx\ddot{\eta}=k^{2}\hat{\eta} \tag{S25}$$

Substituting Eq. S25 into Eq. S23, it can get:

$$\sigma=\frac{(\mu_{2}-\mu_{1})vk-\frac{\gamma d_{\mathrm{L}}^{2}}{12}k^{3}}{\mu_{1}+\mu_{2}} \tag{S26}$$

Taking the first derivative of $\sigma=f(k)$ yields the $k_{\max}$ and $\lambda_{\max}$ at maximum of σ:

$$\lambda_{\max}=\frac{2\pi}{k_{\max}}=\pi\sqrt{\frac{d_{\mathrm{L}}^{2}\gamma}{(\mu_{2}-\mu_{1})v}} \tag{S27}$$

According to Eq. S12, $\sigma>0$, η increases over t, and it gradually evolves into an instable interface-wave and Mvs form. If $\sigma\leqslant 0$, η does not increase over t, and the interface-wave becomes stable, in which no Mvs form. As a result, the increased two-phase interface tension can restrain the interface instability, while the higher displacing velocity can promote the interface instability.

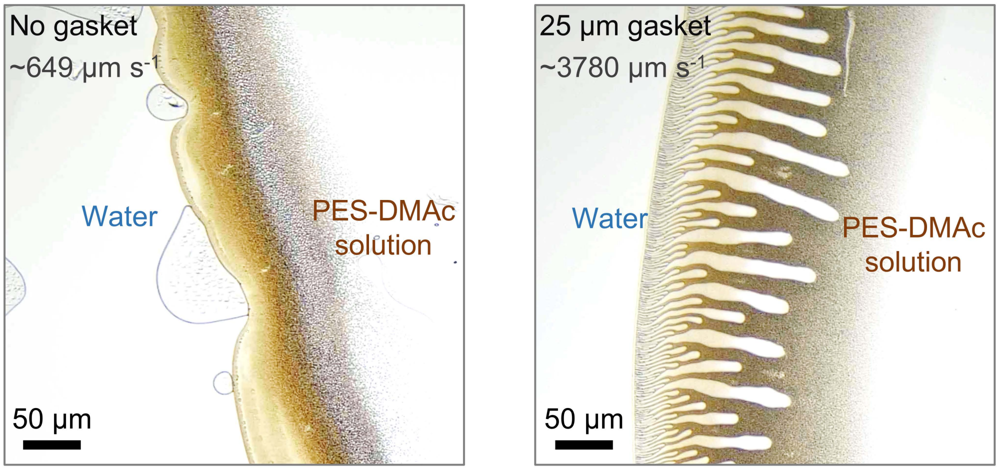

Fig. S12. Microscopic images of polymer precipitation with different nonsolvent impacting velocity. Polymer solution: 20 wt. % PES–DMAc solution. Nonsolvent: water. OC: no gaskets (left), 25 μm gaskets (right). By adjusting the gaskets thickness, the impacting velocity of water could be regulated. By increasing the nonsolvent impacting velocity from ~649 μm s^{-1} to ~3780 μm s^{-1}, the higher proportion of DF/RF was obtained, causing increased interfacial instability. (**Fig. 2f**). That was to say the Mv formation became easier. From **Fig. S12**, as for the PES/DMAc/Water system, the change in the nonsolvent impacting velocity caused the appearance and disappearance of Mvs as well. That phenomenon verified the effectiveness of viscous fingering in clarifying the Mvs formation for different polymer/solvent/nonsolvent systems in NIPS.

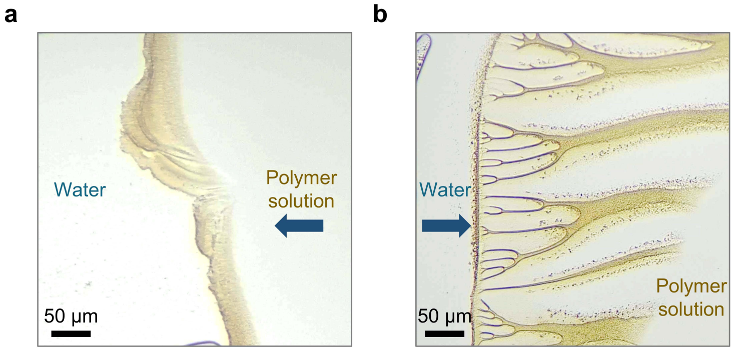

Fig. S13. Microscopic images of polymer precipitation with different modes. (a) The mode of nonsolvent (low viscosity) displacing polymer solution (high viscosity). (b) The mode of polymer solution (high viscosity) displacing nonsolvent (low viscosity). Polymer solution: 5 wt.% PBI–DMAc solution. Nonsolvent: water. OC: no gaskets. According to **Fig. S10**, the low-concentration polymer solution was more likely to form Mvs in NIPS. Thus, we selected a 5 wt.% low-viscosity PBI–DMAc solution. Meanwhile, lower-viscosity water served as the nonsolvent. We fixed one droplet between two glass slides as the displaced liquid, and then added the other liquid as the displacing liquid. As shown in **Fig. S13**, the change in the displacing direction caused the difference in the polymer precipitation micostructure. According to Eq. 3, when the high-viscosity polymer solution displaced the low-viscosity nonsolvent (μ_2-$\mu_1 < 0$), $\sigma < 0$, and the interface-wave was stable (**Fig. 2f**). Under these circumstances, no Mvs formed, consistent with the experimental phenomenon (**Fig. S13a**). As a contrast, when the high-viscosity polymer solution was displaced by the low-viscosity nonsolvent (μ_2-$\mu_1 > 0$), σ was larger than 0 in an interval, and the interface-wave was instable (**Fig. 2f**). Under these circumstances, many Mvs formed (**Fig. S13b**). That comparison further confirmed the Mv formation conformed to the hydrodynamic instability theory.

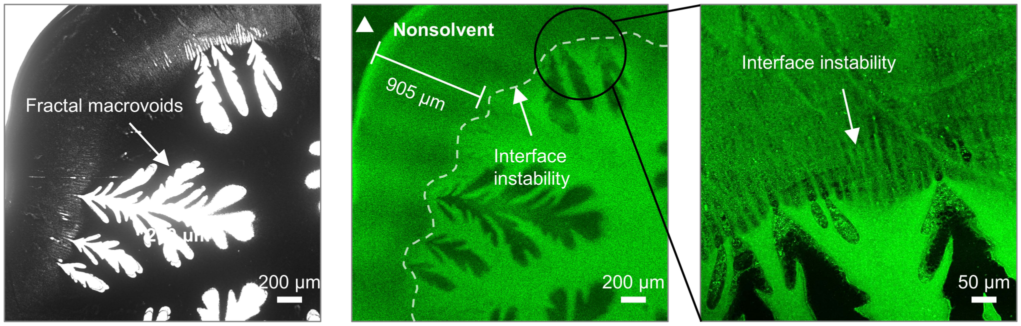

Fig. S14. Visible light (grey) and fluorescence microscopic (green) images of polymer precipitation. Mvs form, following a thick sponge-like layer containing CPs. Polymer solution: 15 wt.% PBI–DMAc solution. Nonsolvent: water. OC: no gaskets. The hydrodynamic instability might not only occur at the interface where the polymer solution and the nonsolvent initially met, but also happen at the front of the ongoing phase separation. From **Fig. S14**, when the thickness of the precipitated Mv-free polymer layer exceeded 905 μm, polymer precipitation rate reduced due to slowing solvent/nonsolvent exchange, leading to ready aggregation of polymer-poor phases to minimize the interface energy. Further, the internal stress easily induced hydrodynamic instability, thereby causing the fractal Mv formation.

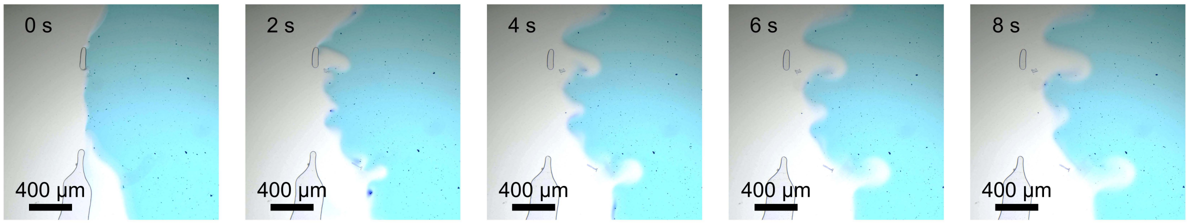

Fig. S15. Microscopic observation for the process of water (colorless) displacing PEG 200 (TB-stained, blue). OC: 50 µm gaskets. Although PEG200 was highly miscible with water, finger-like structures formed at the interface when water displaced PEG200. This phenomenon further corroborated that the Mv formation was attributable to hydrodynamic mechanism

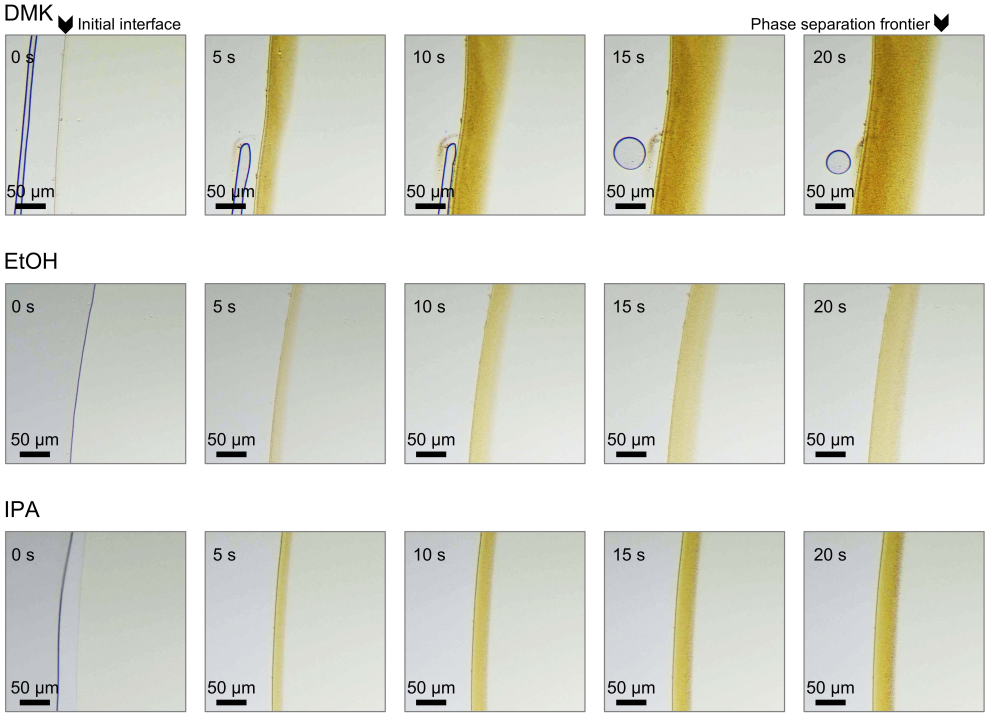

Fig. S16. Microscopic images of CP formation. Polymer solution: 15 wt.% PBI–DMAc solution. OC: no gaskets. In the OC without the gasket, the phase separation interface moved steadily into the interior of the polymer solution. Notably, the phase separation rate induced by different nonsolvents exhibited significant differences, as the critical nonsolvent contents required for polymer precipitation and nonsolvent–solvent exchange rates vary.

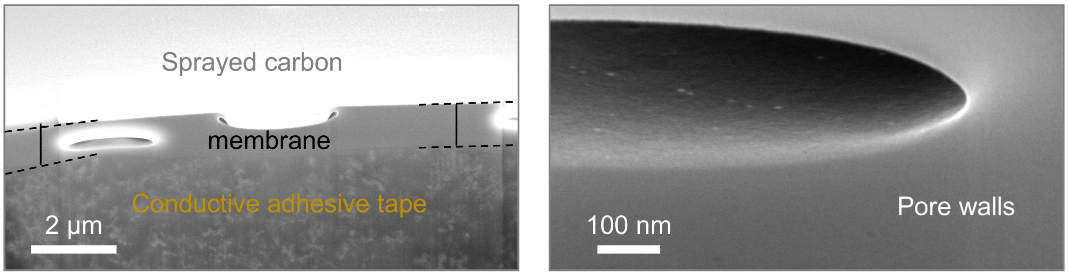

Fig. S17. Cross-sectional images of membranes obtained by FIB-SEM. Polymer solution: 15 wt.% PBI–DMAc solution. Nonsolvent: nHex. Casting thickness: 10 μm. At 50000× magnification (right), no additional micropores was observed adjacent to big pores. Thus, owing to similar shapes, these big pores should be classified as larger-sized CPs. FIB-SEM magnification: right ×3500; right ×50000.

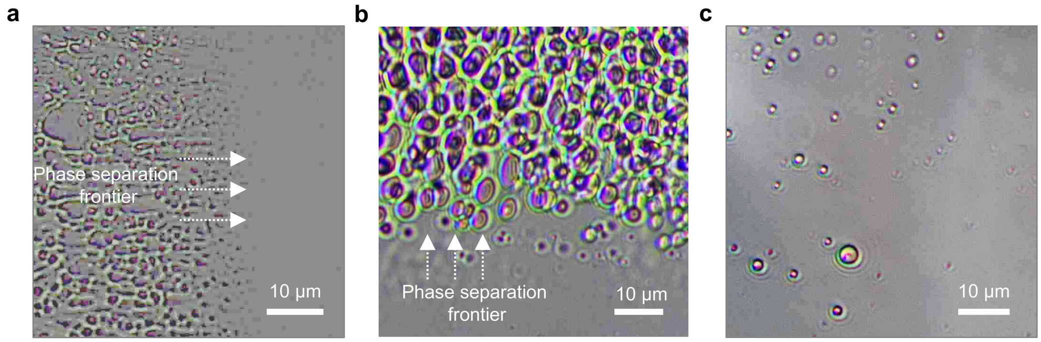

Fig. S18. Microscopic images of polymer-poor phase (i.e., CPs, circular features) at the phase separation frontier. Nonsolvent: (a) water, (b) EtOH, and (c) nHex. Polymer solution: 15 wt.% PBI–DMAc solution. CP formation in NIPS followed nucleation-growth process rather than spinodal demixing. There was an obvious phase separation frontier when water and EtOH were used as the nonsolvent, respectively. As a contrast, when nHex was used as the nonsolvent, the CPs nucleation density was low and no clear phase separation frontier formed.

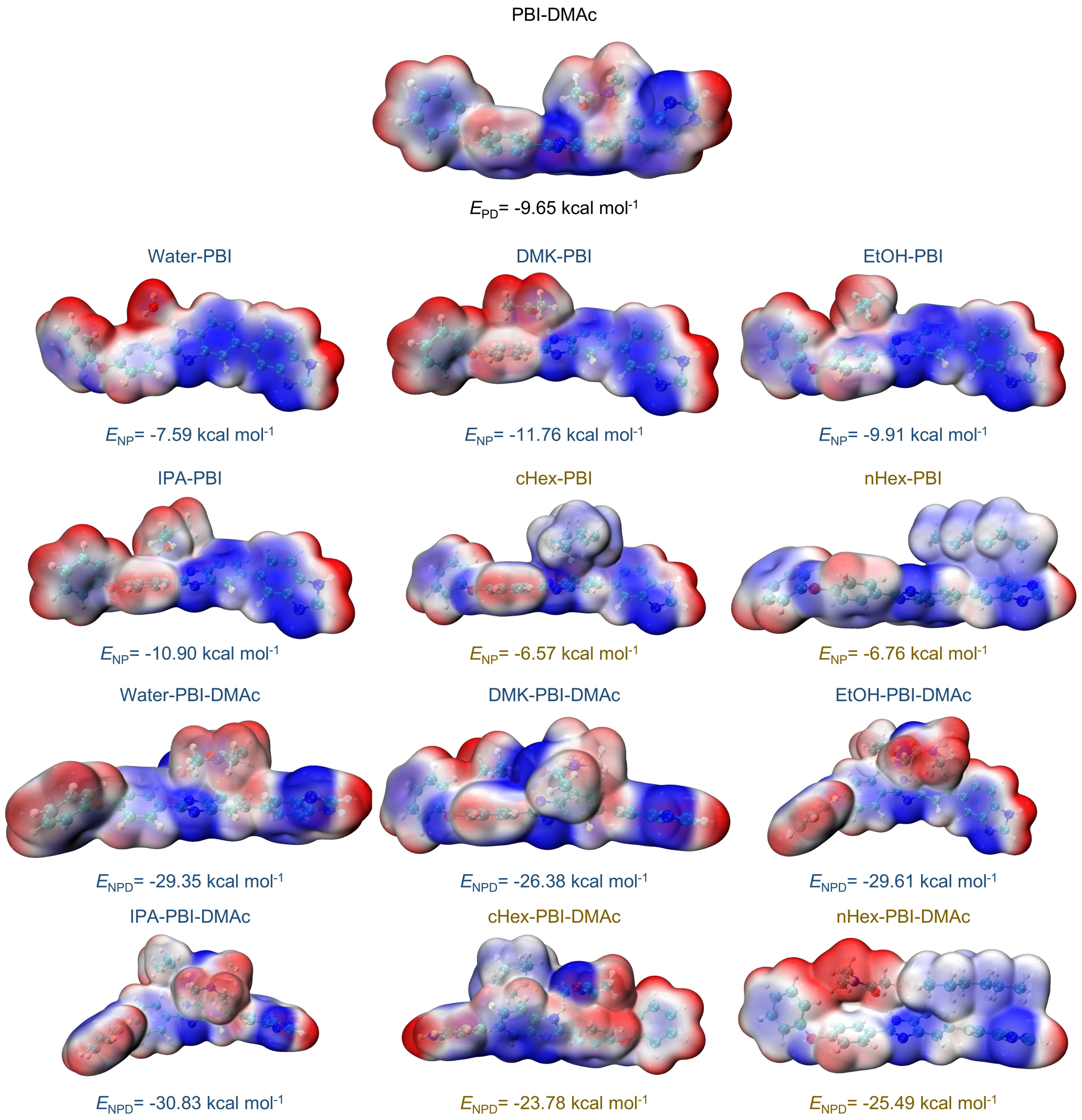

Fig. S19. The interaction energy and electrostatic potential surface between components calculated by DFT. E_{PD} is the interaction energy between PBI unit and DMAc, E_{NP} ithat between nonsolvents and PBI unit, and E_{NPD} is that among nonsolvents, PBI unit, and DMAc. The interactions between Type I nonsolvents and PBI were stronger than that between Type II nonsolvents and PBI. Three-component interaction energies also yielded similar conclusions. Thus, it was necessary to further investigate the correlation between component interactions and CPs.

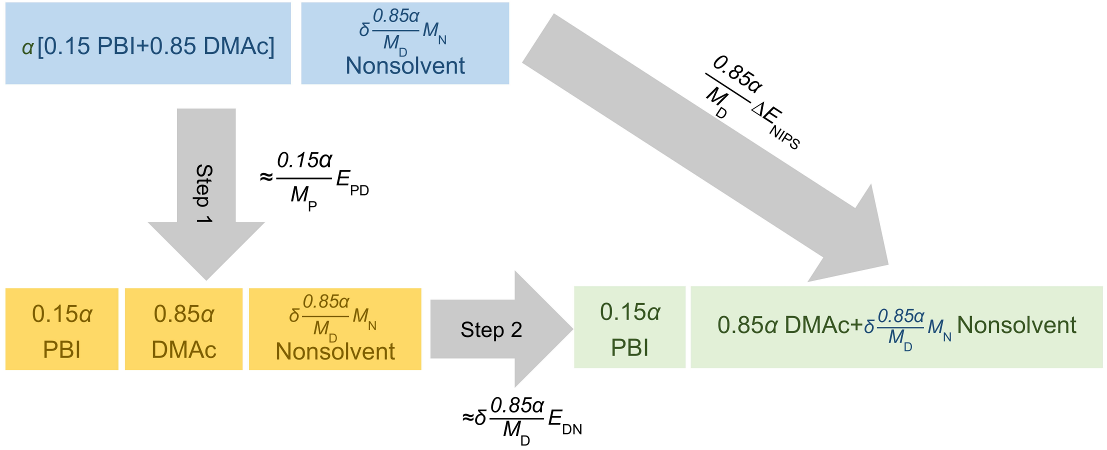

$$\frac{0.85\alpha}{M_D}\Delta E_{NIPS} \approx \frac{0.15\alpha}{M_P}E_{PD} + \delta\frac{0.85\alpha}{M_D}E_{DN}$$

Fig. S20. Schematic diagram of thermodynamic cycle used to roughly estimate energy changes in the NIPS process. α is the mass of the polymer solution. δ is the mole ratio of nonsolvent and DMAc in the cloudy point (Table S6). M_N, M_D and M_P are mole mass of nonsolvents, DMAc and PBI unit, respectively. ΔE_{NIPS} is the energy change in the NIPS per unit mole of DMAc. E_{DN} is the interaction energy between nonsolvents and DMAc. According to the thermodynamic cycle, the energy change in the NIPS process equaled the sum of the energy changes in Step 1 and Step 2. As shown in Table S6, at the cloudy point, the molar amounts of both PBI units and nonsolvents were less than that of DMAc. Thus, the molar amounts of PBI unit and nonsolvent were used to evaluate the energy changes in Step 1 and Step 2, respectively. To eliminate the effect of α, the molar amount of DMAc was used to obtain ΔE_{NIPS}. The summary calculation results were listed in Table S7.

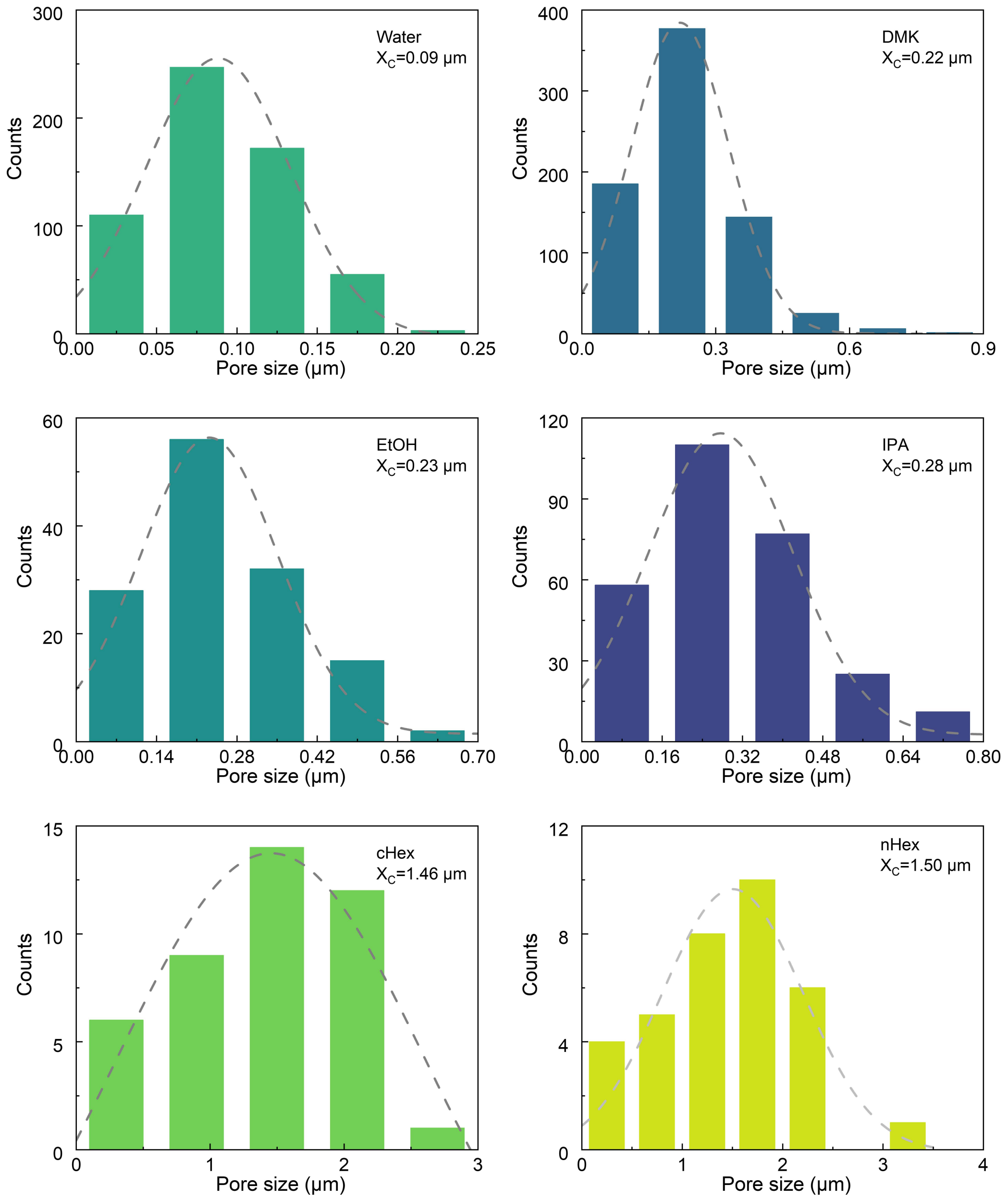

Fig. S21. Statistical distribution diagram of CP sizes prepared in different nonsolvents. Gray line is a Gaussian distribution fitting curve. Polymer solution: 15 wt.% PBI–DMAc solution. Casting thickness: 100 μm.

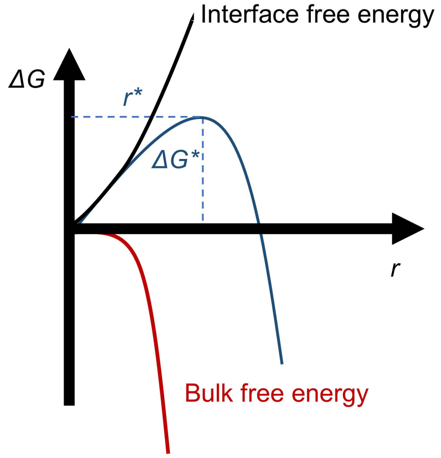

Fig. S22. Relationship between the size of the liquid nucleus (*r*), the interface free energy that needs to overcome for nucleation (black curve), the bulk free energy providing nucleation driving force (red curve), and the total free energy (blue curve, ΔG). Here, r^* denotes the critical size, and ΔG^* represents the nucleation activation energy barrier. According to classical nucleation theory [37], the formation and growth of nucleus are driven by the release of bulk free energy and need to overcome the interface energy associated with increased surface area. ΔG was obtained by the following equation:

$$\Delta G = \frac{4}{3}\pi r^3 \Delta g + 4\pi r^2 \gamma \qquad \text{(S28)}$$

Here, Δg represents the bulk free energy difference between the ternary system at the phase separation and un-separation bulk, and γ denotes the interfacial tension of the nucleus.
As shown in Eq. S22 and S28, ΔG exhibits a maximum value, corresponding to the ΔG^*, with the corresponding nucleus critical size. Therefore, the greater the interface tension, the more difficult it is to nucleate.

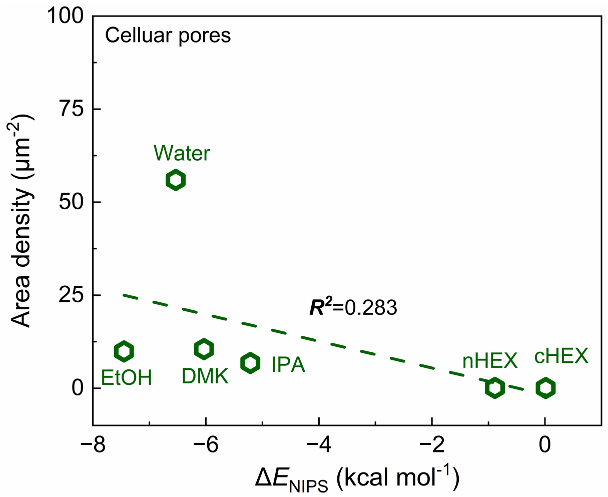

Fig. S23. Correlation between area density of CPs and energy change (ΔE_{NIPS}) in NIPS process. When water was used as the nonsolvent, a significant deviation was observed in **Fig. S23**, thus requiring considering the influence of nonsolvent molecular molar volume.

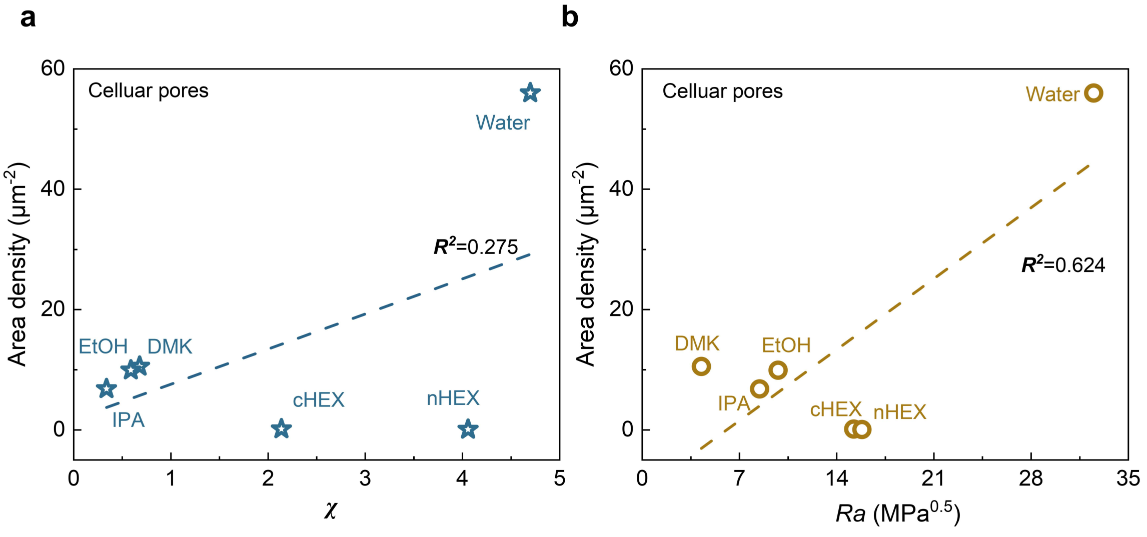

Fig. S24. (a) Correlation between the area density of CPs and the Flory-Huggins interaction parameter (χ) between PBI and nonsolvents. (b) Correlation between the area density of CPs and the solubility distance (Ra) between solvent and nonsolvents, calculated using Hansen solubility parameters. Flory-Huggins interaction parameter (χ) [38] was used to quantitatively characterize the interaction between polymer and nonsolvent, calculated as follows:

$$\chi = \frac{V_m}{RT}(\delta_1 - \delta_2)^2 + 0.34 \tag{S28}$$

where V_m is the molar volume of nonsolvent; δ_1 and δ_2 are the Hildebrand solubility parameters of nonsolvent and polymer, respectively; R is the universal gas constant, and T is the absolute thermodynamic temperature. A smaller χ value indicates a stronger interaction between polymer and nonsolvent, as well as better miscibility between the nonsolvent and the polymer solution. However, the calculated χ value showed an extremely low correlation with the areal density of CPs (R^2=0.275). This aroused because the χ values were clearly inconsistent with the cloud point measurements (Table S6) and DFT calculation results (**Fig. S19**), thus significantly reducing the correlation.

Hansen solubility parameter [38] was employed to predict the interaction between nonsolvent and solvent, with the calculation methods given as follows:

$$\delta^2 = \delta_D^2 + \delta_P^2 + \delta_H^2 \tag{S29}$$

$$Ra = \sqrt{4(\delta_{D1} - \delta_{D2})^2 + (\delta_{P1} - \delta_{P2})^2 + (\delta_{H1} - \delta_{H2})^2} \tag{S30}$$

where δ, δ_D, δ_P, and δ_H represent the total solubility parameter, dispersion component, polar component, and hydrogen-bonding component, respectively. Subscripts 1 and 2 denote two different molecules, and *Ra* is the Hansen distance between two molecules.

Ra quantifies the similarity and miscibility between nonsolvent and solvent (i.e., a smaller *Ra* indicates stronger miscibility). Correlating *Ra* with the areal density of CPs yielded a slightly improved correlation, yet the R^2 was only 0.624. This was because the *Ra* also failed to agree well with the nonsolvent solubility in solvent (Table S4). For instance, water and DMAc were miscible in all proportions, dissolved rapidly, and exhibited a significant exothermic behavior upon mixing, but they showed the largest *Ra*.

The calculated values of both χ and *Ra* are listed in Table S8.

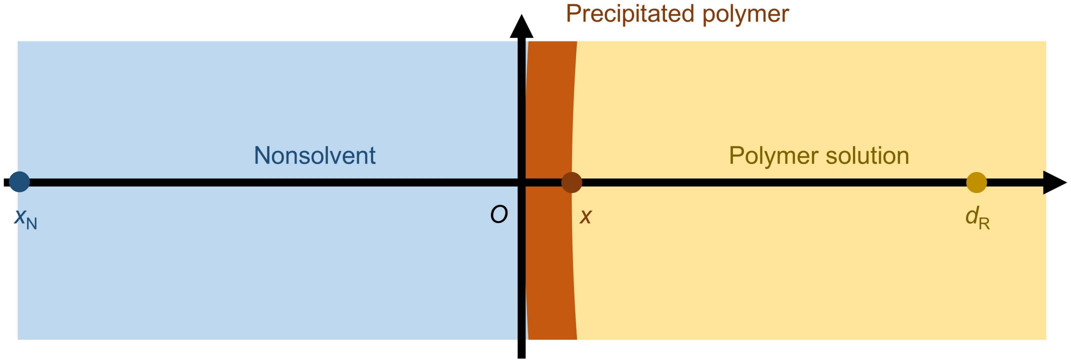

Fig. S25. Cartesian coordinate diagram for Fick's diffusion equation in the OC. Here, O is the initial boundary point between polymer solution and nonsolvent, x movement distance of the phase separation frontier, d_R radius of the polymer solution droplet, and x_N nonsolvent spreading length. For $x < 200$ µm, both d_R and x_N exceed 0.5 cm, i.e., $d_R/x_N > 20x$. In this case, the phase separation frontier can be approximately considered to be relatively straight. Based on that, the double infinite diffusion condition in the Cartesian coordinate system is approximately satisfied:

$$C\left(x>0,0\right)=0,\ C\left(x<0,0\right)=C_0 \tag{S31}$$

$$C(d_R,\ t\geq 0)=0,\ C(x_N,\ t\geq 0)=C_0 \tag{S32}$$

Where, C_0 refers to the molarity of pure nonsolvents.

According to Eq. S31 and S32, the change of nonsolvent molarity ($C(x, t)$) approximately satisfies the Fick's equation as follows:

$$\frac{C(x,t)}{C_0}=0.5erfc(\frac{x}{2\sqrt{Dt}}) \tag{S33}$$

Where, $erfc$ is the residual error function, D diffusion coefficient. x and t are the distance and time in the system, respectively.

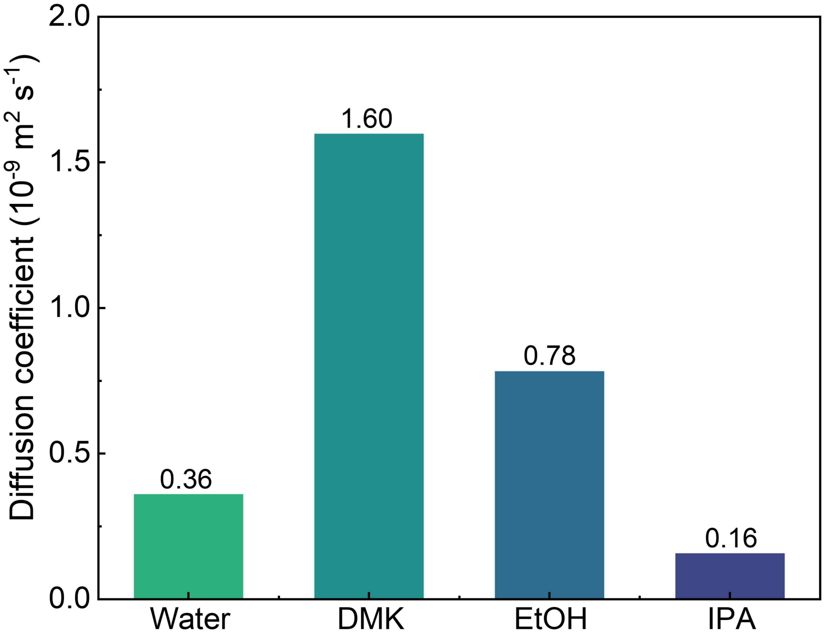

Fig. S26. Diffusion coefficients of different nonsolvents in the 15 wt.% PBI–DMAc solution. C_0 can be calculated from density and molar mass. $C(x,t)$ at the phase separation frontier can be calculated based on the cloud point test results (Table S6). By substituting the value of $1–2C/C_0$ into the numerical table of erf(X), the value inside the parentheses (Defined as X) on the right side of the equal sign of Eq. S33 can be obtained. After substituting the slope k in **Fig. 3f**, D can be calculated as follows:

$$D = \frac{k}{4X^2} \tag{S34}$$

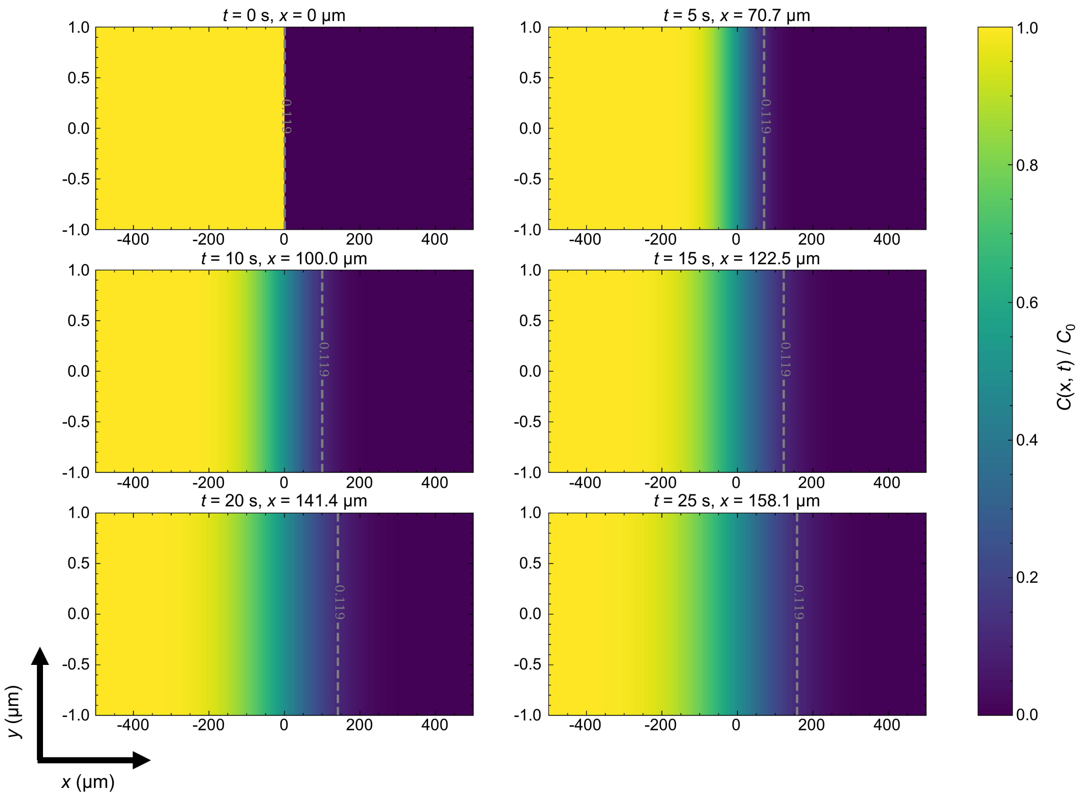

Fig. S27. Python-simulated membrane formation kinetics of water-induced phase separation. C (x, t) and C_0 is the molarity of water in the system and that of pure water, respectively. Gray dashed line: phase separation frontier. Parameters in the Python program: D = 0.359×10^{-9} (diffusion coefficient of the water in the 15 wt.% PBI–DMAc solution) and c_target = 0.119 (C/C_0 at the phase separation frontier).

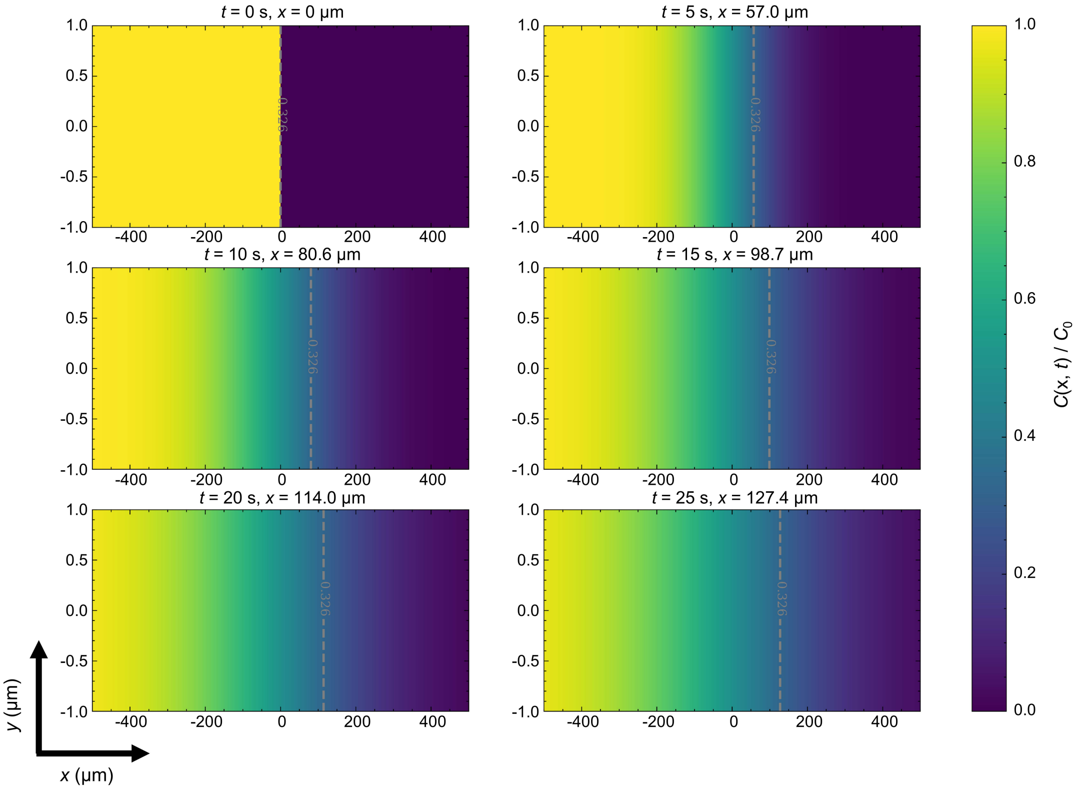

Fig. S28. Python-simulated membrane formation kinetics of DMK-induced phase separation. C (x, t) and C_0 is the molarity of DMK in the system and that of pure DMK, respectively. Gray dashed line: phase separation frontier. Parameters in the Python program: D = 1.597×10^{-9} (diffusion coefficient of the DMK in the 15 wt.% PBI–DMAc solution) and c_target = 0.326 (C/C_0 at the phase separation frontier).

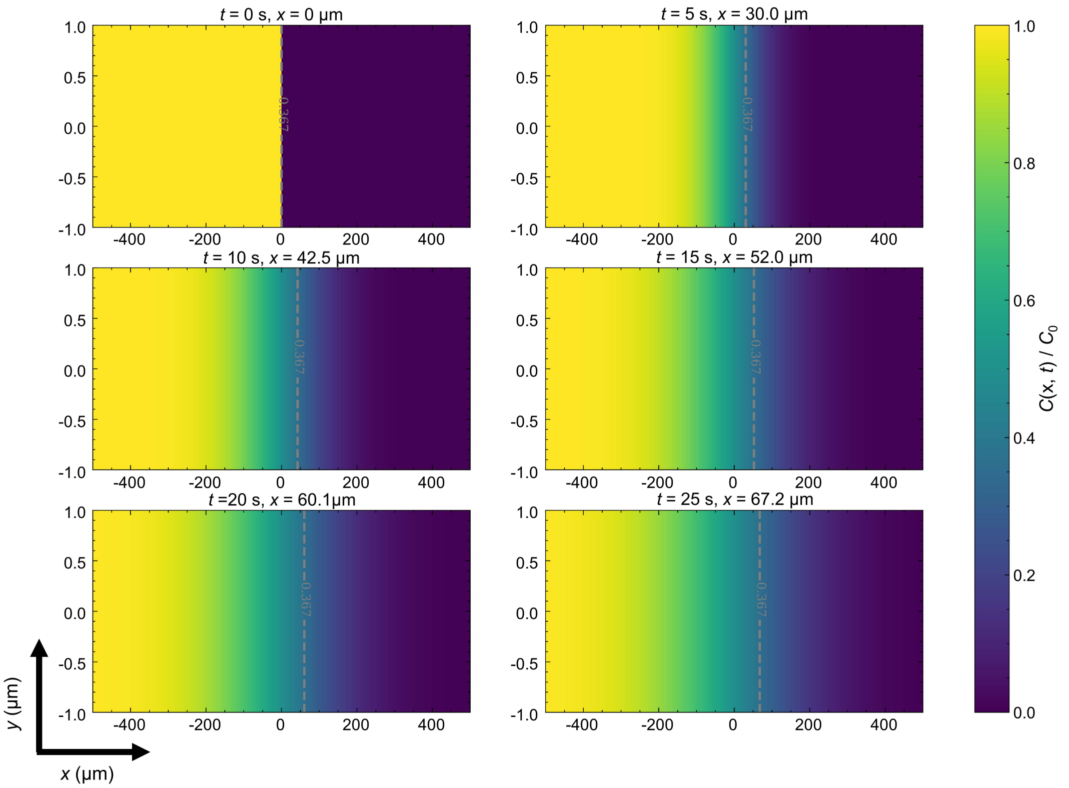

Fig. S29. Python-simulated membrane formation kinetics of EtOH-induced phase separation. C (x, t) and C_0 is the molarity of EtOH in the system and that of pure EtOH, respectively. Gray dashed line: phase separation frontier. Parameters in the Python program: D = 0.781×10^{-9} (diffusion coefficient of the EtOH in the 15 wt.% PBI–DMAc solution) and c_target = 0.367 (C/C_0 at the phase separation frontier).

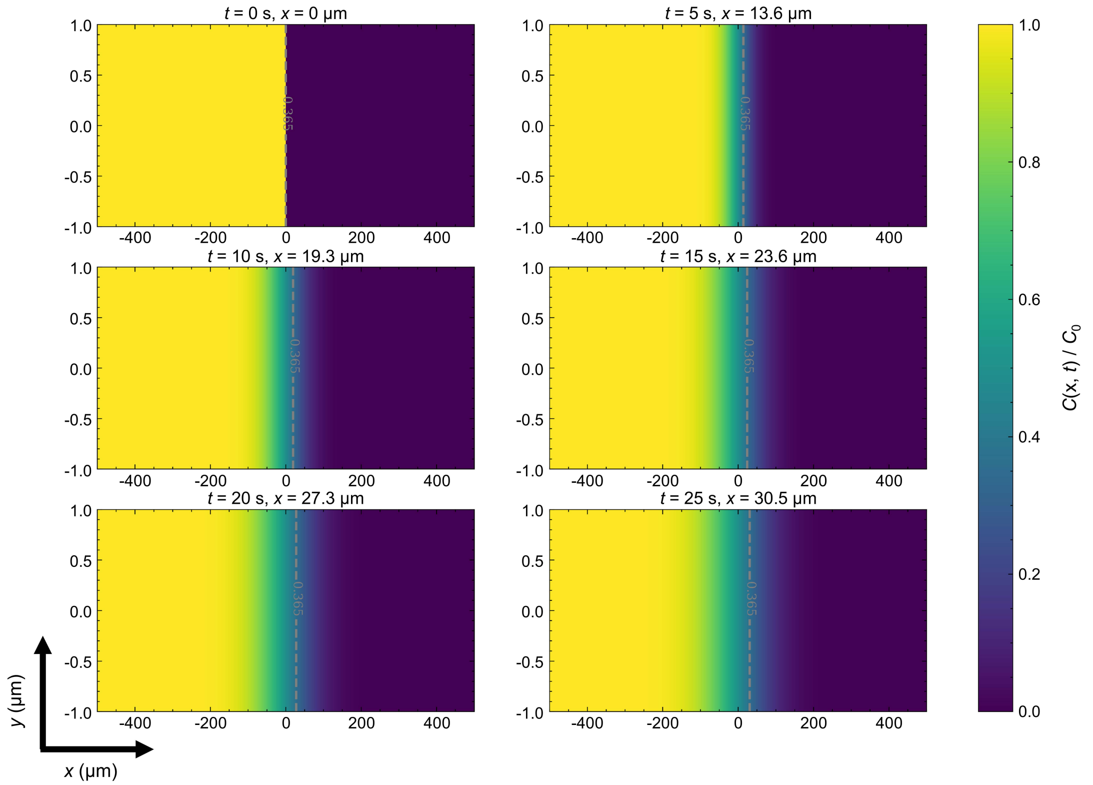

Fig. S30. Python-simulated membrane formation kinetics of IPA-induced phase separation. C (x, t) and C_0 is the molarity of IPA in the system and that of pure IPA, respectively. Gray dashed line: phase separation frontier. Parameters in the Python program: D = 0.156×10^{-9} (diffusion coefficient of the IPA in the 15 wt.% PBI–DMAc solution) and c_target = 0.365 (C/C_0 at the phase separation frontier). From **Figs. S26–S29**, the phase separation rate does not correspond to the diffusion coefficient of the nonsolvent in the ternary system. Therefore, the C/C_0 of nonsolvents within the system should also be considered.

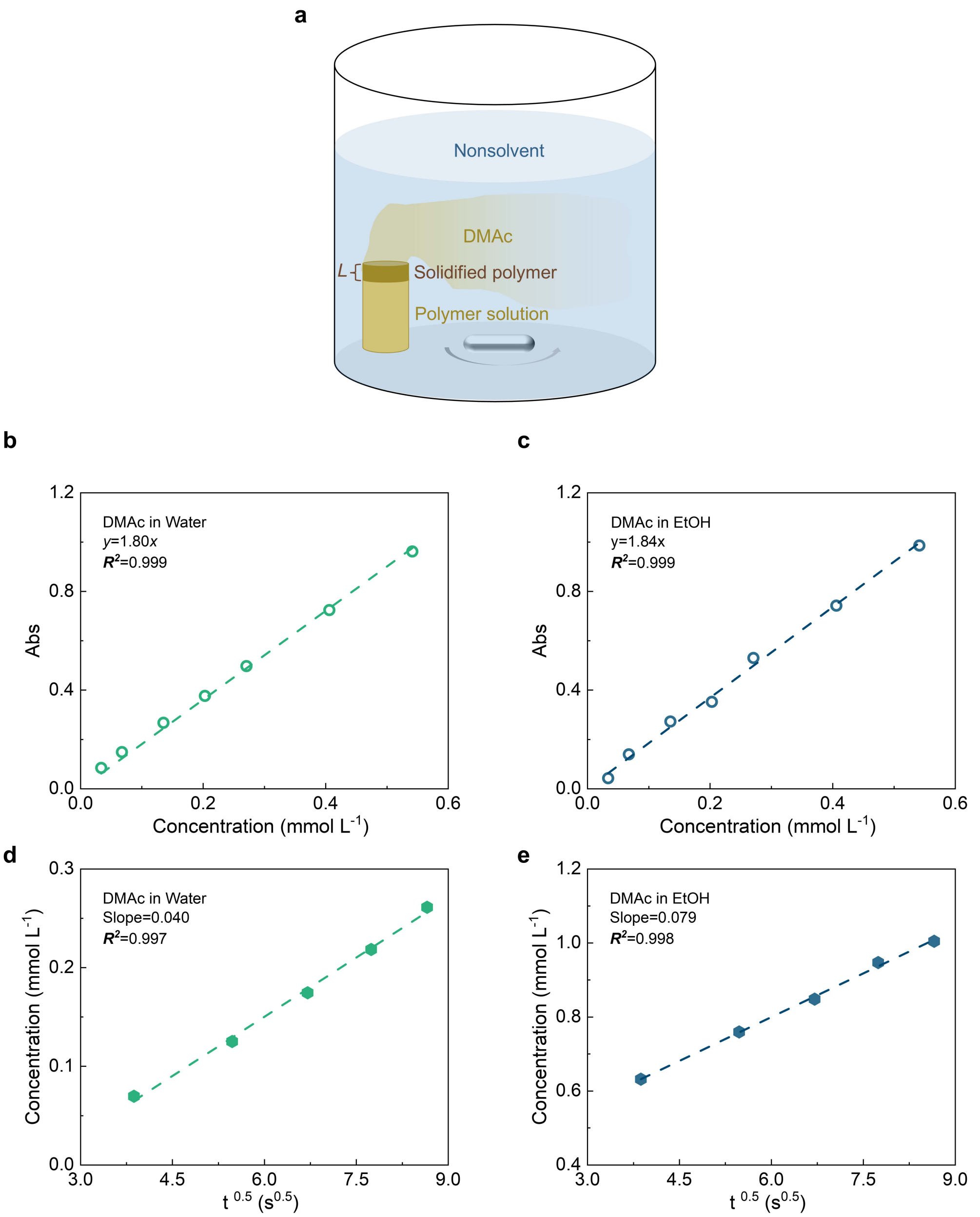

Fig. S31. (a) Schematic of the device for measuring mass transfer of DMAc during the NIPS process. *L* denotes the length of the solidified polymer (brown), the yellow denotes the polymer solution, and the blue denotes the nonsolvent. Magnetic stirring was used to weaken concentration polarization in the beaker. UV-Vis spectroscopy standard curves of DMAc (at 218 nm) in (b) water and (c) EtOH. Plots of DMAc concentration in (d) water and (e) EtOH versus the square root of time ($t^{0.5}$) during the NIPS process. Assuming that stirring eliminates concentration polarization in the nonsolvent outside the polymer solution and solidified polymer, the following equation holds according to Fick's Law:

$$C_{\mathrm{DMAc}}(t) = \int_0^t J dt = DS\int_0^t \frac{C_{\mathrm{P}} - C_{\mathrm{DMAc}}(t)}{L} dt \qquad \text{(S35)}$$

Here, C_{DMAc} is the DMAc concentration in the nonsolvent beaker, t time, J flux of DMAc released in beaker, D diffusion coefficient of DMAc in the phase separation region, S cross-sectional area of polymer solution tube, C_P concentration of DMAc at critical point of polymer precipitation, and L thickness of the solidified polymer.

Since $C_P >> C_{DMAc}$, C_P is a constant and according to **Fig. 3f**, it can be known that L=n$t^{0.5}$+m (n and m are both constants), substituting it into Eq. S35 can get:

$$C_{DMAc} = \mathrm{K}t^{0.5} + \mathrm{B} \tag{S36}$$

Here, K and B are both constants.

Therefore, **Fig. S30** proved that the mass transfer of DMAc was Fick's diffusion behavior. Moreover, the diffusion rate (slope in **Fig. S30e**) of DMAc in EtOH was higher than that of DMAc in water (slope in **Fig. S30d**), which was consistent with the law in **Fig. S25**. This further corroborated that membrane formation kinetics could not be explained only by the nonsolvent-solvent exchange.

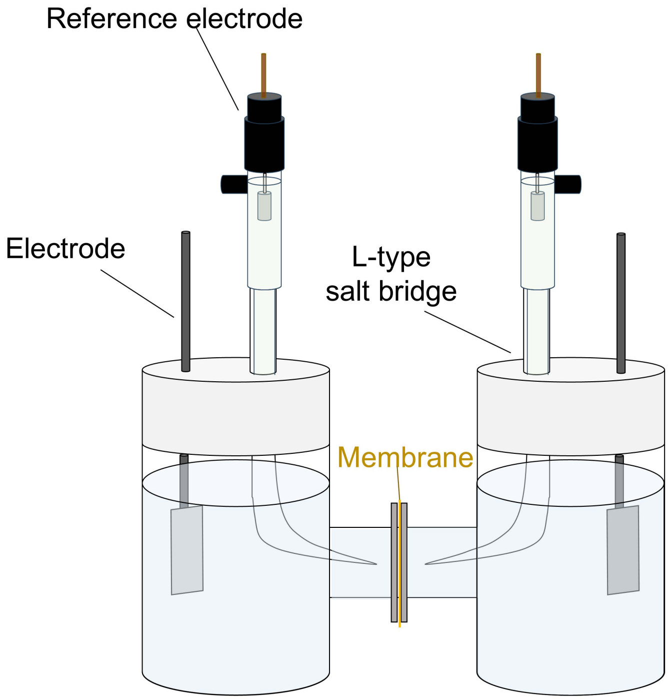

Fig. S32. Schematic diagram of an H-type glass device for ion selectivity and area resistance measurement.

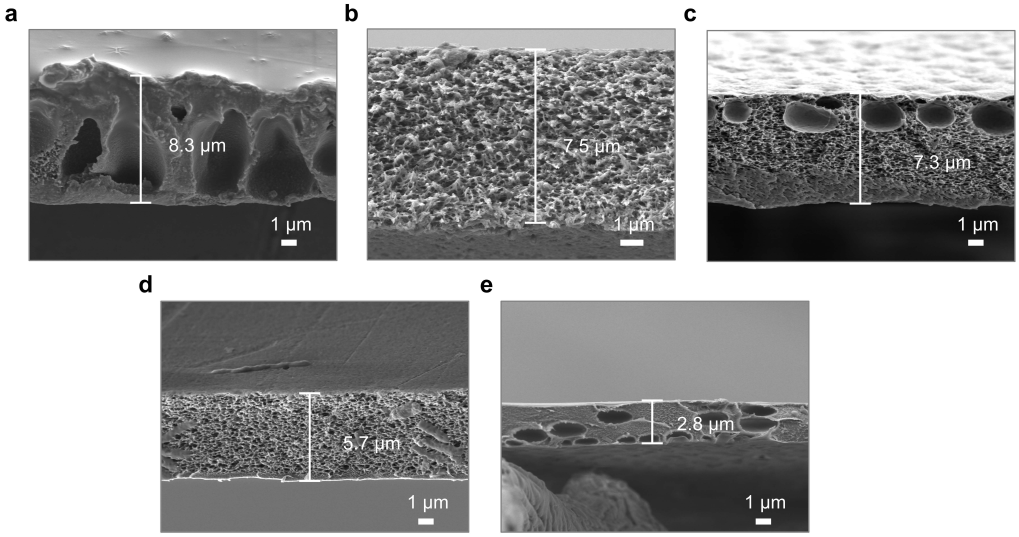

Fig. S33. Cross-sectional SEM images of thin porous membranes prepared in different nonsolvents. (a) Water, (b) DMK, (c) EtOH, (d) IPA and (e) cHex. Polymer solution: 15 wt.% PBI–DMAc solution. Casting thickness: 10 μm. SEM magnification: (a) ×6000; (b) ×9000; (c) ×6000; (d) ×6000; (e) ×6000.

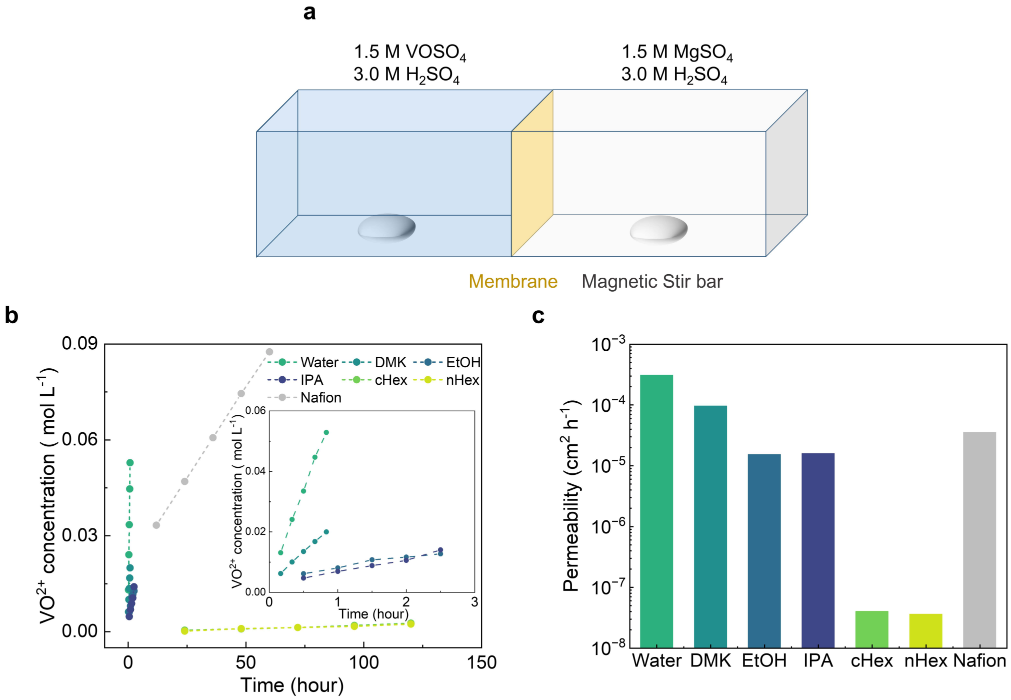

Fig. S34. (a) Schematic diagram of the VO^{2+} permeation device. (b) Plot of VO^{2+} concentration in the $MgSO_4$ solution cell as a function of time. (c) VO^{2+} permeability of thin porous membranes prepared in different nonsolvents. Polymer solution: 15 wt.% PBI–DMAc solution. Casting thickness: 10 μm.

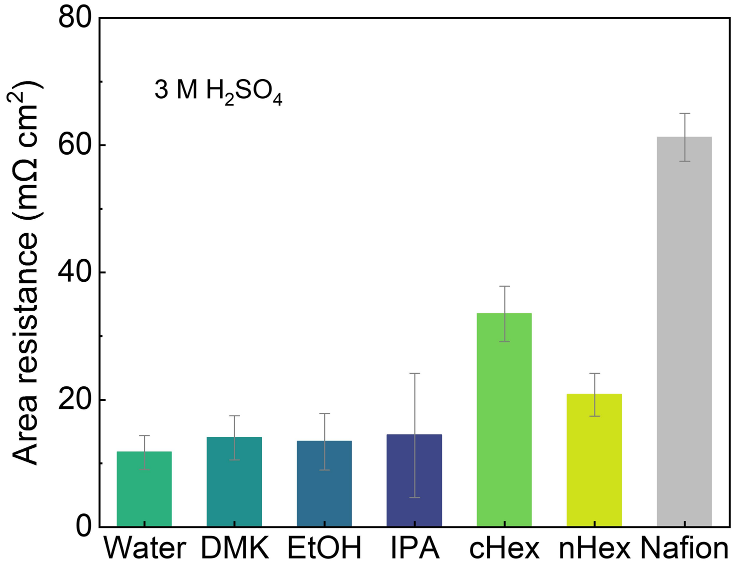

Fig. S35. Area resistances of thin porous membranes prepared in different nonsolvents and Nafion 212 (abbreviation as Nafion in figure). Polymer solution: 15 wt.% PBI–DMAc solution. Casting thickness: 10 µm. Test solution: 3 M H_2SO_4.

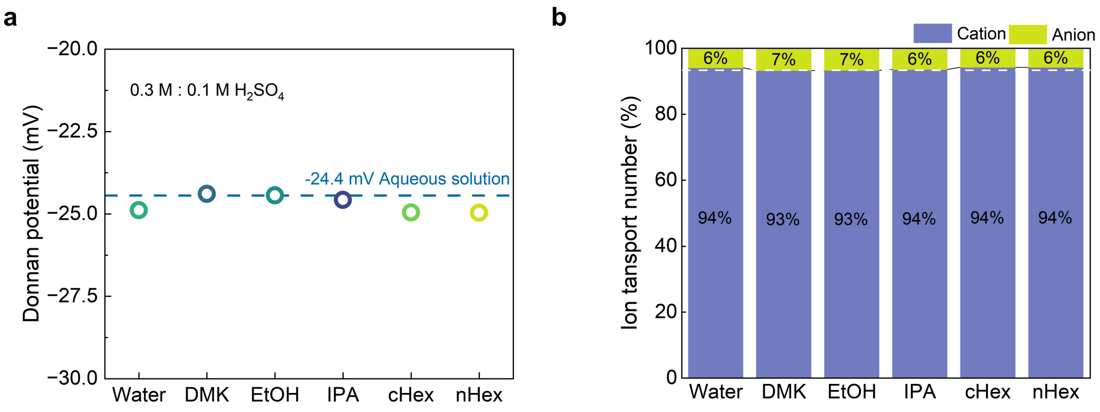

Fig. S36 (a) Transmembrane Donnan potential and (b) ion transport number of thin porous membranes prepared in different nonsolvents. The blue line in (a) represents the Donnan potential formed by the migration of anions and cations (H^+) in the aqueous solution, and the white line in (b) denotes the ion transport numbers of anions and cations (H^+) in aqueous solution. Higher cation transport numbers indicated that all membranes tended to conduct protons. Polymer solution: 15 wt.% PBI–DMAc solution. Casting thickness: 10 μm. Test solution: 0.3 M and 0.1 M H_2SO_4.

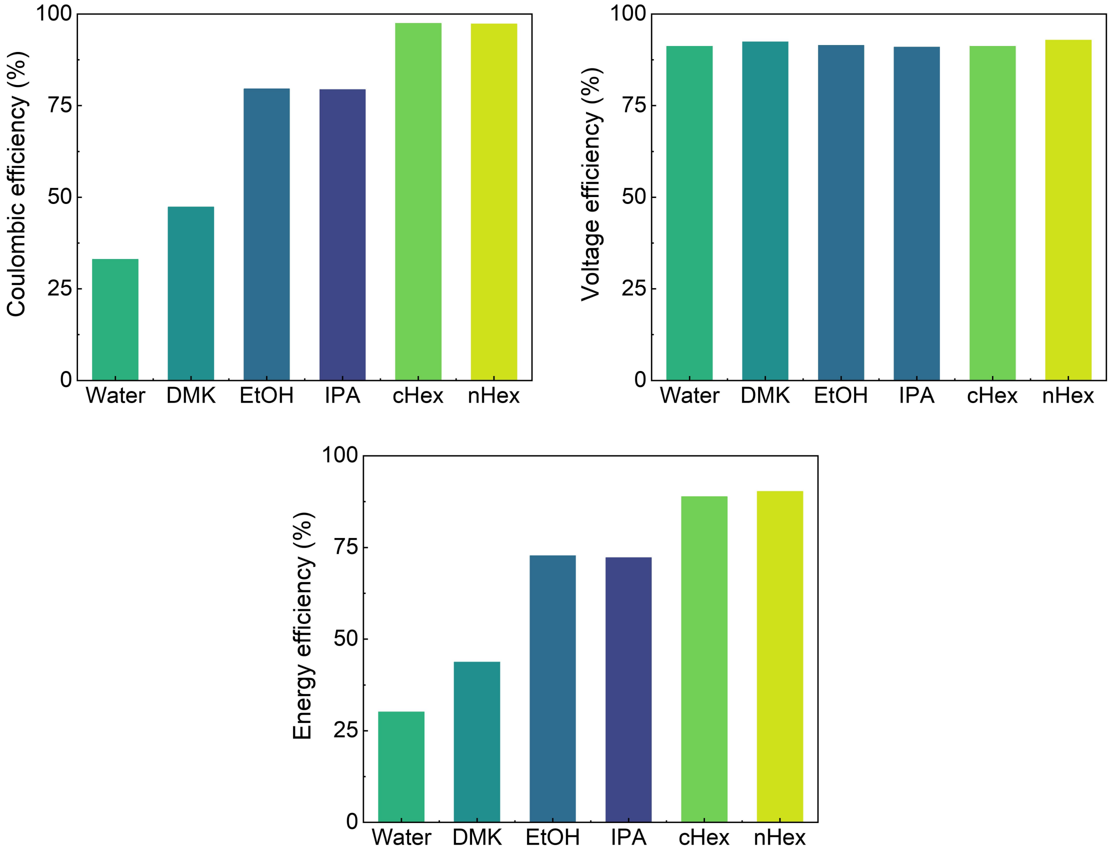

Fig. S37. Coulombic efficiency (CE), voltage efficiency (VE) and energy efficiency (EE) of VFBs assembled with the thin porous membranes prepared in different nonsolvents. Polymer solution: 15 wt.% PBI–DMAc solution. Casting thickness: 10 μm.

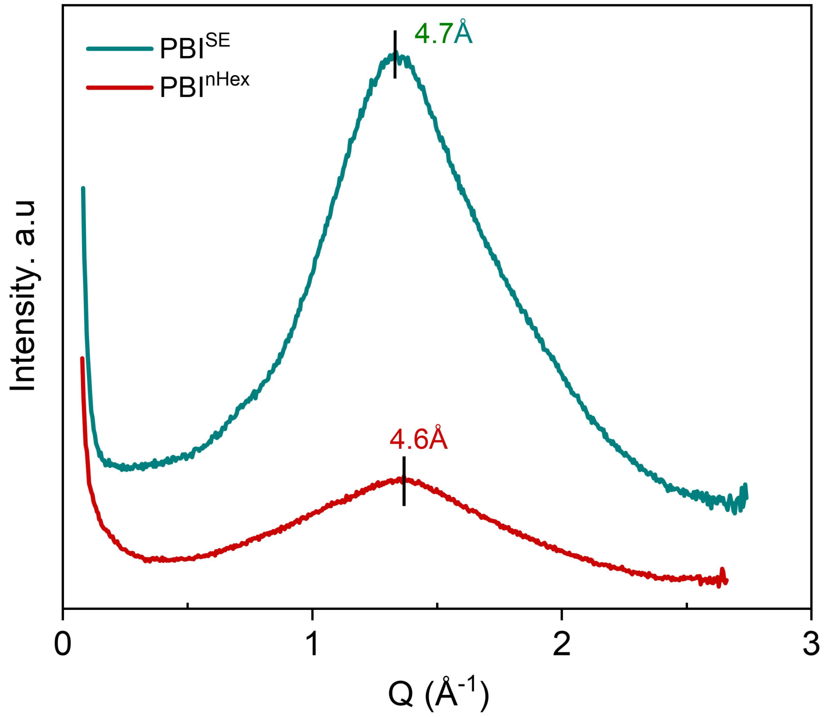

Fig. S38. WAXS patterns of thin porous membrane prepared in nHex (PBI^{nHex}) and PBI membranes prepared by the solvent evaporation method (PBI^{SE}). The WAXS result indicated that the stacking mode of polymer segments of PBI^{nHex} were comparable to that of PBI^{SE}. The peak of PBI^{nHex} stacking void, at 4.6 Å [39], falls between the size of hydrated proton (4.1 Å) and vanadium ions (>8.1 Å) [39], thus endowing it with high ion selectivity.

SUPPLEMENTARY TABLES

Table S1. Summary of microstructures of polymeric membranes prepared by the NIPS approach reported in previous works. M+C represents macrovoids (M) surrounded by cellular pores (C).

Polymer	Solvent	Nonsolvent	Pores	Ref
PES	DMAc<br>NMP<br>DML<br>2P	Water	M+C	[40]
PVDF	NMP	Water	M+C	[41]
	DMF	Water	M+C	[42]
		95% EtOH<br>+5%Water	C	
PVDF+PU	DMK+DMF	Water	M+C	[43]
FD-silica/Si-PU	Ethyl acetate	EtOH	C	[44]
PVA+PVP	DMF	Water	M+C	[45]
PAA	NMP	Water	M+C	[46]
PVC	DMAc	Water	M+C	[47]
CTA+PEG	DMSO	Water	M+C	[48]
PES+PVP	DMAc	Water	M+C	[49]
PVDF	DMAc		M+C	
PBI	DMAc	Water	M+C	[50]
		Water	M+C	[51]
		NaCl+Water	C	
PES+SPEEK	DMAc	Water	C	[52]
		EtOH	M+C	
		IPA	C	
PAN	DMF	Water	M+C	[53]
PAN+PVP	DMF	Water	M+C	[54]

Abbreviations: polyvinylidene difluoride (PVDF), polyurethane (PU), fluorinated silica (FD-silica), silicone-modified polyurethane (Si-PU), polyvinyl alcohol (PVA), polyvinylpyrrolidone (PVP), poly(acrylic acid) (PAA), polyvinyl chloride (PVC), cellulose triacetate (CTA), percutaneous endoscopic gastrostomy (PEG), sulfonated poly(ether ether ketone) (SPEEK), polyacrylonitrile (PAN), 1-Methyl-2-pyrrolidone (NMP), dimethyllactamide (DML), 2-pyrrolidone (2P), N, N-dimethylformamide (DMF), dimethyl sulfoxide (DMSO).

Table S2. Summary of microstructures of polymeric membranes prepared by the VIPS approach reported in previous works. The typical VIPS approach only produces CPs for all different polymers and nonsolvent vapors (water as most commonly used vapor). C represents CPs. RH is relative humidity and RT is room temperature.

Polymer	Solvent	Vapor	Pores	Ref
PVDF	NMP	70%RH and 30°C	C	[55]
	DMSO			[56]
PMIA	DMAc +LiCl	50°C and 90%RH<br>70°C and 90%RH	C	[57]
PVDF	NMP	25°C and 70%RH<br>45°C and 70%RH<br>65°C and 70%RH	C	[58]
SBS+CA	THF	RT and EtOH vapor	C	[59]
PVDF-HFP	DMK	30°C and 96%RH<br>30°C and EtOH vapor<br>30°C and Ammonia vapor<br>30°C, 80% RH and Ammonia vapor	C	[60]
Si-TPU 4-6 + CTPU	DMF+THF	RT and 100%RH	C	[61]
Fe_3O_4@PDA@PIL+ PEG-2000/PES	DMSO	25°C and 80%RH	C	[62]
PEGylated PSF	DMAc	25°C and 95%RH	C	[63]
PES+ $Ni(OH)_2$ + Graphite powder	DMAc	25°C and 60/70/80/90%RH	C	[64]

Abbreviations: poly(m-phenylene isophthalamide) (PMIA), poly(styrene-block-butadiene-block-styrene) (SBS), cellulose acetate (CA), poly(vinylidene fluoride-co-hexafluoropropylene) (PVDF-HFP), thermoplastic polyurethane (TPU), silicon-based TPU (Si-TPU), commercial TPU (CTPU), polydopamine (PDA), poly(ionic liquid) (PIL), 1,4-epoxybutane (THF).

Table S3. Summary of microstructures of polymeric membranes prepared by the thermally induced phase separation (TIPS) approach reported in previous works. Similar with VIPS, the TIPS approach only produces CPs. C represents CPs.

System	Pores	Ref
Chitosan/Water/EtOH	C	[65]
PS/PVME/toluene	C	[66]
PMP/cHex	C	[67]
PHBV/3-hydroxyvalerate/1,4-dioxane	C	[68]
PVB/PEG400	C	[69]

Abbreviations: polystyrene (PS), poly(vinyl methyl ether) (PVME), poly(4-methyl-1-pentene) (PMP), poly(hydroxybutyrate-co-hydroxyvalerate) (PHBV), polyvinyl butyral (PVB).

Table S4. Maximum solubility of different nonsolvents in the solvent (2.5 g DMAc) at room temperature.

Nonsolvent	Maximum solubility (g)
Water	Any mass
DMK	Any mass
EtOH	Any mass
IPA	Any mass
cHex[a]	Any mass
nHex[a]	0.71

[a] The dissolution rate of cHex and nHex in DMAc is very slow, and there is a clear interface between the two.

Table S5. Estimation of the slit width (d_L) between two glass slides in OC without gaskets.

Polymer solution	No	d_a (cm)	d_b (cm)	d_R (cm)	d_L (µm)	$\bar{d}_L$ (µm)
20 wt.% PES–DMAc	1	0.34	0.338	1.35	14.3	10
	2	0.185	0.162	0.6	10.3	
	3	0.314	0.294	1.5	8.6	
15 wt.% PBI–DMAc	1	0.219	0.184	0.8	9.2	
	2	0.151	0.182	0.55	9.1	
	3	0.195	0.209	0.85	7.3	

Table S6. The results of the cloud point test for 2.5 g of 15 wt.% PBI–DMAc solution. δ_N: nonsolvent moles at cloudy point. δ: molar ratio of nonsolvent to DMAc at cloudy point. C: nonsolvent concentration at cloudy point. C_0: Pure nonsolvent concentration. *erfinv*: inverse function of error function.

**Nonsolvent**	δ_N **(mmol)**	δ	C **(mol L^{-1})**	C/C_0	***erfinv*** **($1–2C/C_0$)**
Water [a]	19.6	0.80	6.59	0.119	0.835
DMK[a]	15.6	0.64	4.40	0.326	0.240
EtOH [a]	23.5	0.96	6.29	0.367	0.319
IPA[a]	14.7	0.60	4.17	0.318	0.335
cHex	6.96	0.29			
nHex	3.17	0.13			

[a] When a drop of nonsolvent was added, the polymer would obviously precipitate out. Then, the precipitated polymer re-dissolved in DMAc after stirring for a while.

Table S7. The results calculated from the thermodynamic cycle (**Fig. S20**) and Eq. 4.

**Nonsolvent**	E_1 **(kcal)**	E_2 **(kcal)**	ΔE_{NIPS} **(kcal mol^{-1})**	V_m **(L mol^{-1})**	E_V **(kcal L^{-1})**
Water	0.00360	−0.0673	−6.534	0.0181	−361.6
DMK	0.00360	−0.0625	−6.034	0.0735	−82.06
EtOH	0.00360	−0.0763	−7.449	0.0584	−127.6
IPA	0.00360	−0.0545	−5.212	0.0765	−68.13
cHex	0.00360	−0.0122	−0.884	0.106	−8.302
nHex	0.00360	−0.00346	0.0136	0.131	0.104

Table S8. The results calculated from the Flory-Huggins theory (χ) and Hansen solubility parameters (*Ra*) [38].

Nonsolvent	V_m (mL mol^{-1})	δ_D (MPa$^{0.5}$)	δ_P (MPa$^{0.5}$)	δ_H (MPa$^{0.5}$)	δ (MPa$^{0.5}$)	χ	*Ra*
Water	18	15.5	16	42.3	47.8	4.70	32.52
DMK	74	15.5	10.4	7	19.9	0.68	4.27
EtOH	58.5	15.8	8.8	19.4	26.5	0.59	9.79
IPA	76.5	15.8	6.1	16.4	23.6	0.34	8.46
cHex	106	16.8	0	0.2	16.8	2.14	15.24
nHex	131	14.9	0	0	14.9	4.06	15.83
DMAc		16.8	11.5	10.2			
PBI					23.29 [50]		

Movie S1. Video of the NIPS process in OC without gaskets.
Nonsolvent: water. Polymer solution: 15 wt.% PBI–DMAc solution.

Movie S2. Video of the NIPS process in OC with the 25 μm gaskets.
Nonsolvent: water. Polymer solution: 15 wt.% PBI–DMAc solution.

Movie S3. Video of the NIPS process in OC with the 50 μm gaskets.
Nonsolvent: water. Polymer solution: 15 wt.% PBI–DMAc solution.

Movie S4. Video of convection phenomenon when macrovoids formed.
Nonsolvent: water. Polymer solution: 15 wt.% PBI–DMAc solution. OC: with the 50 μm gaskets.

Movie S5. Videos of macrovoid formation observed by fluorescence microscope.
Nonsolvent: water. Polymer solution: 15 wt.% PBI–DMAc solution. OC: with the 50 μm gaskets.

Movie S6. Video of the NIPS process in OC when the high-viscosity fluid (polymer solution) displaced the low-viscosity fluid (nonsolvent).
Nonsolvent: water. Polymer solution: 5 wt.% PBI–DMAc. OC: no gaskets.

SUPPLEMENTARY REFERENCES

1. Loeb S and Sourirajan S. Sea water demineralization by means of an osmotic membrane. In: Gould RF (ed.). *Saline Water Conversion—II.* Washington: ACS Publications, 1962: 117–32.
2. Zsigmondy R and Bachmann W. Ueber neue filter. *Z anorg allg Chem* 1918; **103**: 119–28.
3. Castro AJ. Methods for making microporous products. U.S.: Google Patents; 1981.
4. Ma W, Zhou Z, Ismail N *et al.* Membrane formation by thermally induced phase separation: Materials, involved parameters, modeling, current efforts and future directions. *J Membr Sci* 2023; **669**: 121303.
5. Guillen GR, Pan Y, Li M *et al.* Preparation and characterization of membranes formed by nonsolvent induced phase separation: a review. *Ind Eng Chem Res* 2011; **50**: 3798–817.
6. Tiihonen LV, Bernardo G, Dalgliesh R *et al.* Influence of the coagulation bath on the nanostructure of cellulose films regenerated from an ionic liquid solution. *RSC Adv* 2024; **14**: 12888–96.
7. Tomietto P, Carré M, Loulergue P *et al.* Polyhydroxyalkanoate (PHA) based microfiltration membranes: Tailoring the structure by the non-solvent induced phase separation (NIPS) process. *Polymer* 2020; **204**: 122813.
8. Guillen GR, Ramon GZ, Kavehpour HP *et al.* Direct microscopic observation of membrane formation by nonsolvent induced phase separation. *J Membr Sci* 2013; **431**: 212–20.
9. Yu L, Yang F, Xiang M. Phase separation in a PSf/DMF/water system: a proposed mechanism for macrovoid formation. *RSC Adv* 2014; **4**: 42391–402.
10. Deng X, Zhao Y, Yang F *et al.* Hydrodynamics coupled simulation of fingerlike pore formation during nonsolvent induced phase separation. *Langmuir* 2025; **41**: 13107–18.
11. Tree DR, Iwama T, Delaney KT *et al.* Marangoni flows during nonsolvent induced phase separation. *ACS Macro Lett* 2018; **7**: 582–6.
12. Hopp-Hirschler M, Shadloo MS, Nieken U. Viscous fingering phenomena in the early stage of polymer membrane formation. *J Fluid Mech* 2019; **864**: 97–140.
13. Vanarse VB, Thakur S, Parmar PR *et al.* Linear stability analysis of phase inversion-induced viscous fingering. In: Singh KM, Dutta S, Subudhi S, Singh NK (eds). *Fluid Mechanics and Fluid Power.* Singapore: Springer, 2022, 289–300.
14. Sadrzadeh M and Bhattacharjee S. Rational design of phase inversion membranes by tailoring thermodynamics and kinetics of casting solution using polymer additives. *J Membr Sci* 2013; **441**: 31–44.

15. Jung JT, Kim JF, Wang HH *et al.* Understanding the non-solvent induced phase separation (NIPS) effect during the fabrication of microporous PVDF membranes via thermally induced phase separation (TIPS). *J Membr Sci* 2016; **514**: 250–63.
16. Cai Y, Li J, Guo Y *et al.* In-situ monitoring of asymmetric poly(ethylene-co-vinyl alcohol) membrane formation via a phase inversion process by an ultrasonic through-transmission technique. *Desalination* 2011; **283**: 25–30.
17. Li S, Cui Z, Zhang L *et al.* The effect of sulfonated polysulfone on the compatibility and structure of polyethersulfone-based blend membranes. *J Membr Sci* 2016; **513**: 1–11.
18. Kim HJ, Fouda AE, Jonasson K. In situ study on kinetic behavior during asymmetric membrane formation via phase inversion process using Raman spectroscopy. *J Appl Polym Sci* 2000; **75**: 135–41.
19. Hirosawa K, Iwama T, Sakamoto N *et al.* In situ observation of the structure and dynamics of a polymer solution through nonsolvent-induced phase separation by x-ray photon correlation spectroscopy. *Phys Rev Mater* 2023; **7**: 045605.
20. Pochat-Bohatier C, Werapun W, Bouyer D *et al.* Near-infrared spectroscopy for the quantitative determination of mass transfer and water absorption kinetics by a polymer solution. *J Polym Sci, Part B:Polym Phys* 2010; **48**: 1960–9.
21. Yuan Z, Duan Y, Zhang H *et al.* Advanced porous membranes with ultra-high selectivity and stability for vanadium flow batteries. *Energy Environ Sci* 2016; **9**: 441–7.
22. Zhao Q, Han X, Wang H *et al.* A new digital positron annihilation lifetime spectrometer for a single piece of micron-thickness film. *Nucl Instrum Methods Phys Res A* 2022; **1038**: 166921.
23. Chen Y, Li Y, Luo M *et al.* A non-beam-based Doppler broadening of positron annihilation radiation (DBAR) spectrometer for a single piece of micron-thickness film. *Nucl Instrum Methods Phys Res A* 2024; **1063**: 169286.
24. Shukla A, Peter M, Hoffmann L. Analysis of positron lifetime spectra using quantified maximum entropy and a general linear filter. *Nucl Instrum Methods Phys Res A* 1993; **335**: 310–7.
25. Broeke J, Pérez JMM, Pascau J. *Image processing with ImageJ*. Birmingham: Packt Publishing, 2015, 1-256.
26. Schindelin J, Rueden CT, Hiner MC *et al.* The ImageJ ecosystem: an open platform for biomedical image analysis. *Mol Reprod Dev* 2015; **82**: 518–29.
27. Frisch MJ, Trucks GW, Schlegel HB *et al.* Gaussian 16 Rev. A.03. Wallingford, CT; 2016.
28. Zhao Y and Truhlar DG. The M06 suite of density functionals for main group thermochemistry, thermochemical kinetics, noncovalent interactions, excited states, and transition elements: two new functionals and systematic testing of four M06-class functionals and 12 other functionals. *Theor Chem Acc* 2008; **120**: 215–41.
29. Weigend F and Ahlrichs R. Balanced basis sets of split valence, triple zeta valence and quadruple zeta valence quality for H to Rn: design and assessment of accuracy. *Phys Chem Chem Phys* 2005; **7**: 3297–305.
30. Grimme S, Antony J, Ehrlich S *et al.* A consistent and accurate ab initio parametrization of density functional dispersion correction (DFT-D) for the 94 elements H-Pu. *J Chem Phys* 2010; **132**: 154104.
31. Al-Housseiny TT, Tsai PA, Stone HA. Control of interfacial instabilities using flow geometry. *Nat Phys* 2012; **8**: 747–50.
32. Mirzadeh M and Bazant MZ. Electrokinetic control of viscous fingering. *Phys Rev Lett* 2017; **119**: 174501.
33. Fu X, Cueto-Felgueroso L, Juanes R. Viscous fingering with partially miscible fluids. *Phys Rev Fluids* 2017; **2**: 104001.
34. Cheng X, Chen Y, Li H *et al.* Investigation on capillary flow in tubes with variable diameters. *J Porous Media* 2019; **22**: 1627–38.
35. Saffman PG and Taylor GI. The penetration of a fluid into a porous medium or Hele-Shaw cell containing a more viscous liquid. *Proc R Soc Lond A* 1958; **245**: 312–29.
36. Homsy GM. Viscous fingering in porous media. *Annu Rev Fluid Mech*. 1987; **19**: 271–311.
37. Li J and Deepak FL. In situ kinetic observations on crystal nucleation and growth. *Chem Rev* 2022; **122**: 16911–82.
38. Hansen CM. *Hansen solubility parameters: a user's handbook*. Boca Raton: CRC press, 2007, 284-8.

39. Xia Y, Cao H, Xu F *et al.* Polymeric membranes with aligned zeolite nanosheets for sustainable energy storage. *Nat Sustain* 2022; **5**: 1080–91.
40. Kahrs C and Schwellenbach J. Membrane formation via non-solvent induced phase separation using sustainable solvents: A comparative study. *Polymer* 2020; **186**: 122071.
41. Huang Y-H, Wang M-J, Chung T-S. Development of multifunctional membranes via plasma-assisted nonsolvent induced phase separation. *Nat Commun* 2024; **15**: 1092.
42. Pagliero M, Bottino A, Comite A *et al.* Novel hydrophobic PVDF membranes prepared by nonsolvent induced phase separation for membrane distillation. *J Membr Sci* 2020; **596**: 117575.
43. Javadi O, Fathollahi Zonouz A, Soltanieh M *et al.* PVDF/PU blend membrane separator for lithium-ion batteries via non-solvent-induced phase separation (NIPS). *J Solid State Electrochem* 2021; **25**: 2385–94.
44. Zhang R, Wei J, Tian N *et al.* Facile preparation of robust superamphiphobic coatings on complex substrates via nonsolvent-induced phase separation. *ACS Appl Mater Interfaces* 2022; **14**: 49047–58.
45. Jacquemond RR, Wan CT-C, Chiang Y-M *et al.* Microstructural engineering of high-power redox flow battery electrodes via non-solvent induced phase separation. *Cell Rep Phys Sci* 2022; **3**: 100943.
46. Li Y, Xue J, Zhang X *et al.* Formation of macrovoid-free PMDA-MDA polyimide membranes using a gelation/non-solvent-induced phase separation method for organic solvent nanofiltration. *Ind Eng Chem Res* 2019; **58**: 6712–20.
47. Padilha LF and Borges CP. PVC membranes prepared via non-solvent induced phase separation process. *Braz J Chem Eng* 2019; **36**: 497–509.
48. Xing X-Y, Gu L, Jin Y *et al.* Fabrication and characterization of cellulose triacetate porous membranes by combined nonsolvent-thermally induced phase separation. *Cellulose* 2019; **26**: 3747–62.
49. Lu W, Yuan Z, Zhao Y *et al.* High-performance porous uncharged membranes for vanadium flow battery applications created by tuning cohesive and swelling forces. *Energy Environ Sci* 2016; **9**: 2319–25.
50. Lu W, Yuan Z, Zhao Y *et al.* Advanced porous PBI membranes with tunable performance induced by the polymer-solvent interaction for flow battery application. *Energy Storage Mater* 2018; **10**: 40–7.
51. Qiao L, Zhang H, Lu W *et al.* Advanced porous membranes with tunable morphology regulated by ionic strength of nonsolvent for flow battery. *ACS Appl Mater Interfaces* 2019; **11**: 24107–13.
52. Qiao L, Zhang H, Lu W *et al.* Advanced porous membranes with slit-like selective layer for flow battery. *Nano Energy* 2018; **54**: 73–81.
53. Li Y, Pan G, Zhang Y *et al.* A new method for tailoring the surface pore size and internal pore structure of ultrafiltration membranes without using additives-atomization-assisted nonsolvent induced phase separation method. *Sep Purif Technol* 2023; **304**: 122334.
54. Wan CTC, Jacquemond RR, Chiang YM *et al.* Non-solvent induced phase separation enables designer redox flow battery electrodes. *Adv Mater* 2021; **33**: 2006716.
55. Venault A, Zhou R-J, Galeta TA *et al.* Engineering sterilization-resistant and fouling-resistant porous membranes by the vapor-induced phase separation process using a sulfobetaine methacrylamide amphiphilic derivative. *J Membr Sci* 2022; **658**: 120760.
56. Venault A, Aini HN, Galeta TA *et al.* Using the dimethyl sulfoxide green solvent for the making of antifouling PEGylated membranes by the vapor-induced phase separation process. *J Membr Sci Lett* 2022; **2**: 100025.
57. Huang Z, Chen Y, Han Q *et al.* Vapor-induced phase inversion of poly (m-phenylene isophthalamide) modified polyethylene separator for high-performance lithium-ion batteries. *Chem Eng J* 2022; **429**: 132429.
58. Zhao Q, Xie R, Luo F *et al.* Preparation of high strength poly (vinylidene fluoride) porous membranes with cellular structure via vapor-induced phase separation. *J Membr Sci* 2018; **549**: 151–64.
59. Xiong X, Wang Y, Zhong C. Preparation of an asymmetric membrane via vapor induced phase separation for membrane distillation. *Prog Org Coat* 2023; **181**: 107590.
60. Huo P, Zhong C-T, Xiong X-P. Tailoring morphology of PVDF-HFP membrane via one-step reactive vapor induced phase separation for efficient oil-water separation. *Chin J Polym Sci* 2021; **39**: 610–9.
61. Pan B and Lee KJ. Preparation of thermoplastic polyurethane blend foams with controlled hydrophobicity via vapor induced phase separation. *J Appl Polym Sci* 2023; **140**: 1-8.
62. Wei R, Guo J, Jin L *et al.* Vapor induced phase separation towards anion-/near-infrared-responsive pore channels for switchable anti-fouling membranes. *J Mater Chem A* 2020; **8**: 8934–48.

63. Zhu L-J, Song H-M, Wang G *et al.* Microstructures and performances of pegylated polysulfone membranes from an in situ synthesized solution via vapor induced phase separation approach. *J Colloid Interface Sci* 2018; **515**: 152–9.
64. Dong W, Niu X, Ji X *et al.* Transferring electrochemically active nanomaterials into a flexible membrane electrode via slow phase separation method induced by water vapor. *ACS Sustainable Chem Eng* 2019; **7**: 4295–306.
65. Sochilina AV, Akasov RA, Arkharova NA *et al.* Fabrication of moldable chitosan gels via thermally induced phase separation in aqueous alcohol solutions. *Int J Biol Macromol* 2022; **215**: 501–11.
66. Pathak B, Xavier P, Bose S *et al.* Thermally induced phase separation in levitated polymer droplets. *Phys Chem Chem Phys* 2016; **18**: 32477–85.
67. Zhang T-Q, Hao S, Xiao J *et al.* Preparation of poly (4-methyl-1-pentene) membranes by low-temperature thermally induced phase separation. *ACS Appl Polym Mater* 2023; **5**: 1998–2005.
68. Zeinali R, Khorasani MT, Behnamghader A *et al.* Poly (hydroxybutyrate-co-hydroxyvalerate) porous matrices from thermally induced phase separation. *Polymers* 2020; **12**: 2787.
69. Luo Y, Xu Y, Wang F *et al.* Fabrication of a biconnected structure PVB porous heddle via thermally induced phase separation. *RSC Adv* 2019; **9**: 14599–608.
